# 5^me^CpG Epigenetic Marks Neighboring a Primate-Conserved Core Promoter Short Tandem Repeat Indicate X-Chromosome Inactivation

**DOI:** 10.1371/journal.pone.0103714

**Published:** 2014-07-31

**Authors:** Filipe Brum Machado, Fabricio Brum Machado, Milena Amendro Faria, Viviane Lamim Lovatel, Antonio Francisco Alves da Silva, Claudia Pamela Radic, Carlos Daniel De Brasi, Álvaro Fabricio Lopes Rios, Susana Marina Chuva de Sousa Lopes, Leonardo Serafim da Silveira, Carlos Ramon Ruiz-Miranda, Ester Silveira Ramos, Enrique Medina-Acosta

**Affiliations:** 1 Department of Genetics, School of Medicine, University of São Paulo, Ribeirão Preto, São Paulo, Brazil; 2 Laboratory of Biotechnology, Universidade Estadual do Norte Fluminense Darcy Ribeiro, Campos do Goytacazes, Rio de Janeiro, Brazil; 3 Laboratory of Molecular Genetics of Hemophilia, Institute of Experimental Medicine, National Academy of Medicine, Buenos Aires, Argentina; 4 Department of Anatomy and Embryology, Leiden University Medical Center, Leiden, South Holland, the Netherlands; 5 Laboratory of Animal Morphology and Pathology, Center for Studies and Research in Wildlife, Universidade Estadual do Norte Fluminense Darcy Ribeiro, Campos do Goytacazes, Rio de Janeiro, Brazil; 6 Laboratory of Environmental Sciences, Sector of Studies of Ethology, Reintroduction and Conservation of Wild Animals, Universidade Estadual do Norte Fluminense Darcy Ribeiro, Campos do Goytacazes, Rio de Janeiro, Brazil; 7 Molecular Identification and Diagnostics Unit, Hospital Escola Álvaro Alvim, Campos dos Goytacazes, Rio de Janeiro, Brazil; University of Bonn, Institut of experimental hematology and transfusion medicine, Germany

## Abstract

X-chromosome inactivation (XCI) is the epigenetic transcriptional silencing of an X-chromosome during the early stages of embryonic development in female eutherian mammals. XCI assures monoallelic expression in each cell and compensation for dosage-sensitive X-linked genes between females (XX) and males (XY). DNA methylation at the carbon-5 position of the cytosine pyrimidine ring in the context of a CpG dinucleotide sequence (5^me^CpG) in promoter regions is a key epigenetic marker for transcriptional gene silencing. Using computational analysis, we revealed an extragenic tandem GAAA repeat 230-bp from the landmark CpG island of the human X-linked retinitis pigmentosa 2 *RP2* promoter whose 5^me^CpG status correlates with XCI. We used this *RP2* onshore tandem GAAA repeat to develop an allele-specific 5^me^CpG-based PCR assay that is highly concordant with the human androgen receptor (*AR*) exonic tandem CAG repeat-based standard HUMARA assay in discriminating active (Xa) from inactive (Xi) X-chromosomes. The *RP2* onshore tandem GAAA repeat contains neutral features that are lacking in the *AR* disease-linked tandem CAG repeat, is highly polymorphic (heterozygosity rates approximately 0.8) and shows minimal variation in the Xa/Xi ratio. The combined informativeness of *RP2*/*AR* is approximately 0.97, and this assay excels at determining the 5^me^CpG status of alleles at the Xp (*RP2*) and Xq (*AR*) chromosome arms in a single reaction. These findings are relevant and directly translatable to nonhuman primate models of XCI in which the *AR* CAG-repeat is monomorphic. We conducted the *RP2* onshore tandem GAAA repeat assay in the naturally occurring chimeric New World monkey marmoset (*Callitrichidae*) and found it to be informative. The *RP2* onshore tandem GAAA repeat will facilitate studies on the variable phenotypic expression of dominant and recessive X-linked diseases, epigenetic changes in twins, the physiology of aging hematopoiesis, the pathogenesis of age-related hematopoietic malignancies and the clonality of cancers in human and nonhuman primates.

## Introduction

In eukaryotes, the CpG dinucleotide sequence is distributed sparsely but genome-wide, except in distinct regions termed CpG islands (CGI), in which its density is increased approximately five-fold; these regions generally correspond to promoters [1]. Depending on the methylation state of the carbon-5 position of the cytosine residue, the self-complimentary CpG dinucleotide functions as a genomic signaling sequence for the recruitment of either repressive or permissive histone modification marks, which modulate the chromatin structure into mutually exclusive transcriptionally inactive (silenced) or active configurations, respectively [2]. With the exception of the sites in active promoter regions, nearly 80% of CpG sites in the mammalian genome are in the 5^me^CpG state in somatic cells [2]. Thus, transcriptional silencing correlates positively with the maintenance (in frequency and breadth) of 5^me^CpG in promoter regions.

Gene silencing based on 5^me^CpG marks underlies key cellular processes such as cellular differentiation, cell-, tissue- and embryonic developmental stage-specific gene expression, preservation of chromatin structure and chromosomal integrity, aging of the hematopoietic system, carcinogenesis, random autosomal monoallelic gene expression, parent-of-origin-dependent monoallelic gene expression (genomic imprinting) and X-chromosome inactivation (XCI) [3].

XCI is the stable, (nearly) chromosome-wide transcriptional silencing of either the maternal (^M^X) or the paternal (^P^X) X-chromosome in the inner cell mass of female eutherian mammals [4]. XCI entails selecting (normally at random), targeting and driving either ^M^X or ^P^X in each early stage embryonic female cell into a facultative heterochromatin configuration of sustained transcriptional gene suppression [5], [6].

Overall, XCI ensures monoallelic gene expression in each cell and compensation for dosage-sensitive X-linked genes between females (XX) and males (XY) [7]. In human females, there is extensive variability in X-linked gene expression, with approximately 15% of genes resisting XCI and being expressed from both active X (Xa) and inactive X (Xi) chromosomes and an additional 10% being expressed to varying degrees from some Xi chromosomes [8]. Thus, while most genes on Xi are stably silenced, a discrete yet significant subset of genes escape transcriptional suppression by being excluded from the condensed heterochromatic body of Xi [9]. Escape genes (e.g., active genes on Xi) may exhibit tissue-specific differences in the escape from inactivation [10]. Escape genes have distinct evolutionary implications for sex differences in specific phenotypes [10], [11].

The 5^me^CpG-sensitive restriction endonuclease-based PCR assay targeting the polymorphic trinucleotide tandem CAG repeat (microsatellite, short tandem repeat - STR) in exon 1 of the human androgen receptor (*AR*) gene (MIM 313700) in the Xq12 region, known as the HUMARA assay, is a standard readout method for determining the methylation statuses of alleles on Xa and Xi and is widely used as a marker of X-chromosome activity [12]. The *AR* tandem CAG repeat yields heterozygosity rates of approximately 0.85 worldwide, and it is therefore uninformative in a significant proportion of females. The *AR* tandem CAG repeat genotype is not neutral, with threshold numbers of repeat units being positive and negatively correlated with Kennedy disease (KD [MIM 313200]) [13] and prostate cancer [14], [15], respectively. Moreover, the *AR* CAG-repeat locus is monomorphic in the small nonhuman primate species used in biomedical research [16], which precludes its use in studies of XCI in these important experimental models.

We sought to identify X-linked repeats that are conserved in primates and consist of neutral features to accurately assess the methylation statuses of alleles in Xa and Xi. We aimed to develop a method that is highly concordant with the *AR* disease-linked tandem CAG repeat assay, but with minimal ^M^X/^P^X variation due to lesser *in vitro* replication slippage by Taq polymerase across repeat units greater than triplets. This goal has not been realized to date in either humans or nonhuman primate species.

## Materials and Methods

### Ethics Statement

Samples from human subjects were collected with written informed consent for projects approved by the Ethics Committee of the Faculdade de Medicina de Campos, Brazil (approval code FR-278769); Leiden University Medical Center, the Netherlands (P08.087); Faculdade de Medicina de Ribeirão Preto, Brazil (HCRP 5810/2009); and Institutos de la Academia Nacional de Medicina, Argentina (14/08/2008). The capture of individual marmosets (wild hybrids of *Callithrix jacchus* and *Callithrix penicillata*), confinement in a captive colony, management, care, drawing of biological samples and necropsies were all carried out under authorizations from the Brazilian Chico Mendes Institute for the Conservation of Biodiversity – ICMBio (URL: http://www.icmbio.gov.br/portal/) with license #33965-2 and the Brazilian Institute of the Environment and Renewable Natural Resources - IBAMA (URL: http://www.ibama.gov.br/) with license CGEF AM3301.8101/2013-RJ. The marmoset specimens were taken into captivity in strict accordance with the recommendations of ICMBio as part of a control program for these invasive species. They were previously introduced into an industrial zone belonging to the Brazilian Oil company TRANSPETRO, located in the State of Rio de Janeiro, inhabited by the endangered, native golden lion tamarins (*Leontopithecus rosalia*). The program was licensed by ICMBio and IBAMA because the presence of the marmosets increases the risk of extinction of golden lion tamarins by exposing them to transmissible infectious diseases, predation or limiting-resource competition. The captive colony was founded in the Sector of Studies on the Ethology, Reintroduction and Conservation of Wild Animals (SERCAS, website URL: http://uenf.br/cbb/sercas/) of the Universidade Estadual do Norte Fluminense Darcy Ribeiro, Brazil, as a model for management. Animal management activities were supervised by an IBAMA-licensed, expert investigator (CRRM). The capture, clinical and laboratory examinations and handling of animals were conducted essentially as previously reported [17]. No marmoset specimen was euthanized to obtain tissue for this study. Marmoset peripheral blood samples (50 µL) were drawn into EDTA during routine examination of confined animals. Samples (3–5 mm^3^) of muscle, liver, brain and skin/hair tissues were strictly taken from the frozen remains of necropsies carried out by a licensed veterinarian (LSS) that were exclusively performed on specimens that died of natural causes during the process of adapting to confinement including failure to thrive, wasting syndrome and/or nematode infestation. Care was taken to alleviate suffering, and measures were implemented according to IBAMA guidelines for the well-being of wildlife and the recommendations of the Guide for the Care and Use of Laboratory Animals of the Universidade Estadual do Norte Fluminense Darcy Ribeiro, Brazil.

### Subjects

To determine heterozygosity rates and allele frequencies, we genotyped two population subsets, each consisting of sixty healthy, unrelated women from Brazil and the Netherlands. To analyze the correlations between random or non-random X-inactivation patterns and the *RP2*-extragenic GAAA repeat or the *AR* exonic CAG repeat (HUMARA assay), we genotyped a third subset of fifty unrelated women who had known HUMARA-based methylation profiles (e.g., Xa/Xi ratios). We genotyped four healthy male donors as a control for methylation-sensitive restriction enzyme activity. To demonstrate the power of *RP2*-extragenic GAAA repeats in discriminating Xa from Xi in heterozygous female carriers of an X-linked recessive defect that manifests due to non-random (skewed) X-inactivation, we genotyped four confirmed heterozygous carriers of hemophilia A. Two of these individuals were conventional, non-symptomatic carriers who screened positive for *F8* intron 22 inversions via inverse shifting-PCR [18] and for random X-inactivation via the *AR* CAG repeat assay [12]. The other two were heterozygous carriers of missense and frameshift mutations in factor VIII domains A1 and B, respectively. They were screened through conformational sensitive gel electrophoresis [19] and direct sequencing and presented with a severe hemophilia A phenotype due to extremely skewed XCI. For the assessment of marmosets, we genotyped necropsy tissues from twenty-two adult subjects (fourteen females and eight males from different social groups).

### Cells

The THP-1 cell line was cultured in RPMI-1640 medium, 10% fetal bovine serum, penicillin/streptomycin, 10 mM HEPES, 1 mM sodium pyruvate and 50 µM 2-mercaptoethanol [20].

### DNA and RNA extraction

Human genomic DNA was extracted from either peripheral blood or mouth epithelial cells (swabs) utilizing a commercial Illustra blood genomic Prep Mini Spin kit (GE Healthcare, Little Chalfont, UK) [21]. Genomic DNA from blood samples from female carriers of the *F8* defect, the Dutch population subset and the marmoset necropsy tissues (blood, muscle, liver, brain and skin) was extracted via phenol-chloroform and ethanol precipitation [22]. Total cellular RNA from human nucleated blood cells and the THP-1 cell line was extracted using TRIzol reagent (Invitrogen, Carlsbad, CA, USA).

### Digestion with methylation-sensitive restriction enzymes

Genomic DNA (500 ng) was digested with *Hpa*II (Invitrogen, Carlsbad, CA, USA), *Bst*UI and *Hha*I (New England Biolabs, Ipswich, MA, USA) for 6 h at 37°C (*Hpa*II and *Hha*I) or 60°C (*Bst*UI), or was mock-digested without the restriction enzymes. The final volume of the reaction mixture was 10 µL. Throughout the methylation-based PCR assays, 5^me^CpG-sensitive restriction endonuclease activity was assessed by genotyping DNA from four healthy males (not shown).

### Analysis of allele-specific methylation

DNA genotyping was carried out in quantitative fluorescence polymerase chain biplex reactions (QF-PCR) in approximately 50 ng of digested or undigested DNA using 0.8 µM (*AR*) and 1.2 µM (*RP2*) of each primer pair (Table S1). The thermal cycling conditions were as follows: 95°C for 11 minutes (1 cycle); 94°C×1 min, 59°C×1 min and 72°C×1 min (28 cycles); and 60°C×60 min (1 cycle) in a Gene Amp PCR system 9700 (Applied Biosystems, Foster City, CA, USA). The allele profiles and areas under the curves for each allele were determined in an ABI 310 Prism Genetic Analyzer (Applied Biosystems). The data were analyzed with GeneScan Analysis 3.7 and Genotyper 3.7 software (Applied Biosystems). Fluorescent peak areas representing true alleles were normalized for the occurrence of stutter products using the approach outlined in the literature [23]. The degree of association between the percentages of the Xi/Xa referred by the methylation statuses at the *RP2* GAAA onshore and *AR* CAG repeat loci across women with varying extents of random and non-random XCI was determined by calculating the Spearman correlation coefficient, CI95% and *p* value and visualized with a scatterplot using Graph Pad Prism 5.0.

### Reverse transcription-PCR (RT-PCR)

Samples of 500 ng of total RNA were digested using 1 U of DNAse I (Invitrogen) at room temperature for 15 min and then inactivated by the addition of 1 µl of EDTA (25 mM) and incubation at 65°C for 5 min in a final volume of 10 µL. The DNase I-treated RNA was reverse transcribed to single-stranded cDNA using a High Capacity cDNA Reverse Transcription Kit (Applied Biosystems) according to the manufacturer's protocol. To test for possible transcription spanning the *RP2* GAAA repeat, the primer pair used for QF-PCR typing was employed on target cDNA samples (diluted 10-fold) from nucleated blood cells and the THP-1 cell line. As a positive control, cDNA samples were tested for *GAPDH* expression using the primer sequences shown in Table S1. These primers align to three different locations in reference genomic sequences: *GAPDH* (chr12∶6646089-6646308) and two pseudogenes, *GAPDHP63* (chr6∶80663360-80663489) and *GAPDHP1* (chrX:39647022-39647151). In *GAPDH*, the primers anneal to exons 5 and 6 (the RNA-specific cDNA product is 130-bp in length). In all experiments, mock RT-PCR assays (without Reverse Transcriptase) were included.

### Conservation of the *RP2*-extragenic GAAA repeat in nonhuman primates

The extent of conservation of the GAAA repeat-containing locus in nonhuman primates was investigated computationally using the MegaBLAST search algorithm [24] with the *in silico*-generated human PCR amplimer as the query reference sequence, followed by multiple sequence alignment of the target regions in the Molecular Evolutionary Genetics Analysis (MEGA) stand-alone program [25].

## Results

### Experimental strategy

To ensure success in the identification of highly polymorphic candidate repeat loci, we applied a combined comprehensive computational and empirical strategy consisting of mining the *Homo sapiens* chromosome X GRCh37.p5/hg19 primary reference genome assembly [26] for repeats that fulfill all of the following criteria: (*i*) tetranucleotides or pentanucleotides with at least twelve repeat units and a match percentage >90 according to Tandem Repeat Finder [27] (alignment parameters of 2, 7 and 7 for matches, mismatches and indels, respectively); (*ii*) mapping outside of exons and pseudoautosomal regions [24]; (*iii*) mapping <300-bp from or residing within landmark CpG islands [1] relevant to genes expressed only from Xa (e.g., escape genes excluded) [8]; and (*iv*) the occurrence of at least one 5^me^CpG-sensitive restriction endonuclease site within 300-bp of the tandem repeat. Matching these criteria should improve the base-calling precision of templates and the measurement of true alleles by effectively limiting Taq polymerase stuttering (the magnitude of stuttering decreases as the repeat unit length increases [28], [29]), and allow to achieve the power of informativeness of the *AR* disease-linked CAG repeat assay regarding the methylation statuses of X-chromosomes [12] (*AR* does not escape XCI [8], and the informativeness of repeats on X correlates with the number of perfect tandem repeat units [29], [30]). The real power of this combined approach for predicting highly polymorphic STR loci in promoter regions is its direct applicability to available X-chromosome sequences of any mammalian species.

### Chromosomal and physical map positions and sequence features of the novel locus

**Figure 1:**
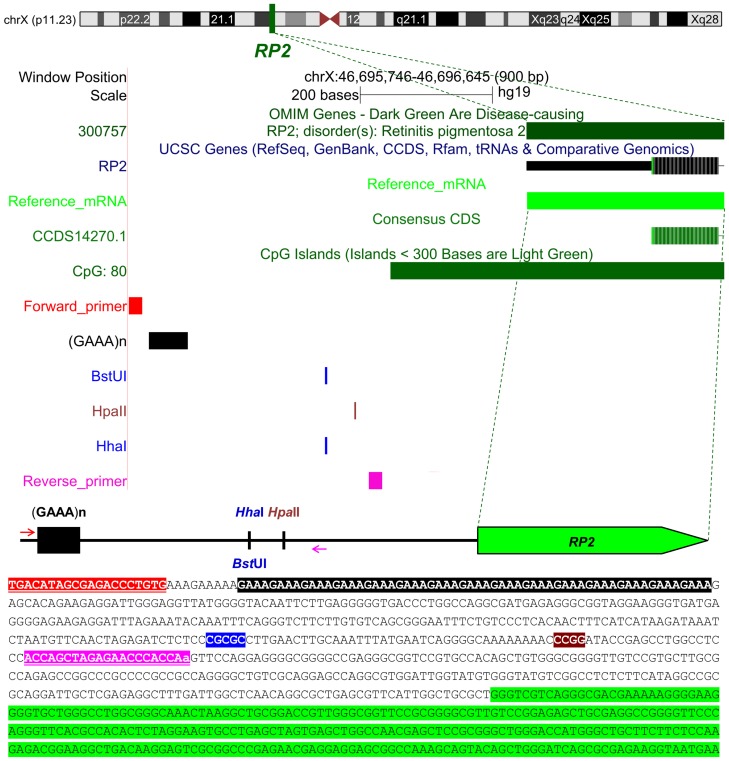
Chromosomal and physical map positions and sequence features of the locus encompassing the *RP2* onshore tandem GAAA repeat. The composite image above the DNA sequence is based on screenshots generated using the UCSC Genome Browser (http://genome.ucsc.edu) [49], with the *RP2* onshore tandem GAAA repeat-containing region viewing coordinates chrX:46,695,746-46,696,645 (GRCh37.p5/hg19 primary reference assembly of human X-chromosome; NC_000023.10), centered on the landmark CpG island of the *RP2* promoter. The GAAA repeat element maps within the Xp11.3. The presented features (from top to bottom) are annotated tracks for OMIM genes, UCSC Genes (RefSeq, GenBank, CCDS, Rfam, tRNAs & Comparative Genomics), reference mRNA, CpG and the tandem (GAAA)n repeat. The line drawing above the DNA sequence represents the physical map of the target locus, with the *RP2* 5′coding region highlighted in light green. The locations of the forward and reverse primer sequences used for genotyping the *RP2* onshore tandem GAAA repeat are highlighted in red and pink, respectively. The tandem GAAA repeat sequence is highlighted in black with white symbols. The 5^me^C-sensitive restriction endonuclease recognition sites analyzed in the XCI experiments are highlighted in blue and brown in white symbols.

The endeavor rendered only one, albeit suitable, repeat: a tetranucleotide repeat element (physical location chrX:46695765-46695834) near *RP2* (MIM 300757) (Figure 1), the gene corresponding to X-linked retinitis pigmentosa 2 (MIM 312600), which maps to Xp11.3 [31] and does not escape XCI [8], [31]. Using the alignment parameters 2, 7 and 7 for matches, mismatches and indels, respectively, Tandem Repeats Finder marks the repeat unit as AAAG. However, comparison of three public reference genomic sequences showed that the alleles consist of multiple copies of the GAAA repeat unit (Figure S1). Henceforth, we refer to this repeat element as GAAA to indicate the physical location of the GAAA repeat-containing allele in the GRCh37.p5/hg19 primary reference assembly of the human X-chromosome.

The GAAA repeat is positioned -582, -598 or -630-bp (upstream) of known transcription start sites of *RP2* (Figure S2). The element maps on shore, 230-bp upstream of the *RP2* CpG island (Genomic coordinates NC_000023.10 Reference GRCh37.p5 Primary Assembly X:46695995-46696984), a landmark that exhibits differential methylation [1], displaying increased methylation on Xi in 46, XX and reduced methylation in 45, X females [32]. The *RP2* onshore tandem GAAA repeat is therefore positioned approximately 20 Mb upstream of the *AR* disease-linked, exonic CAG repeat, which maps to Xq12.

The *RP2* onshore tandem GAAA repeat does not overlap with *RP2* cDNAs, known transcription factor binding sites (Figure S2), cap analysis gene expression promoters (Figure S3) or microRNA precursors (Figure S2) that are predicted or annotated in public repositories (see Web Resources) [24], [33].

### Reverse transcription-PCR across the *RP2* onshore tandem GAAA repeat

**Figure 2:**
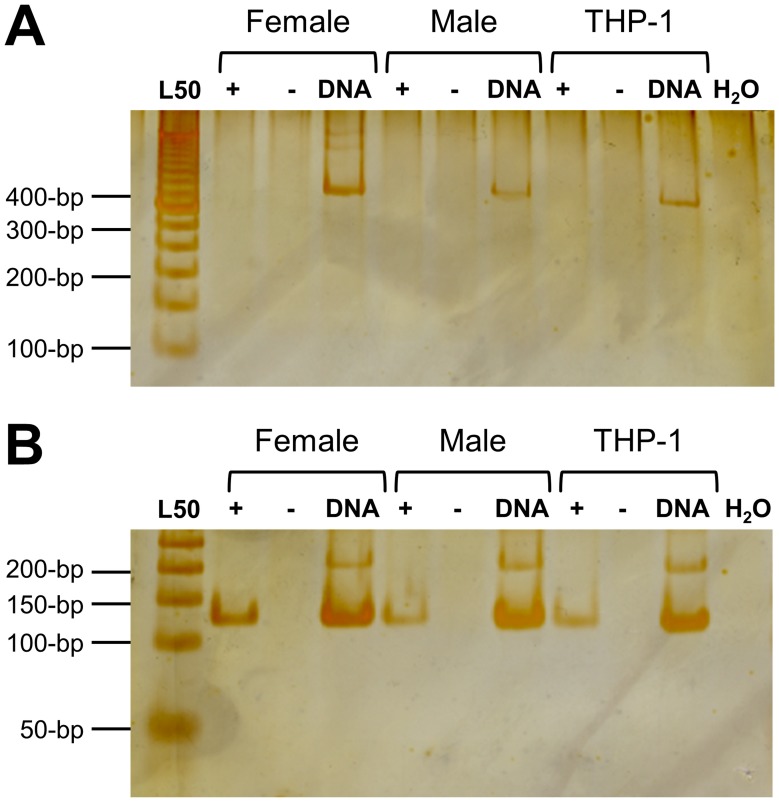
Reverse transcription-PCR across the GAAA repeat-containing region. *RP2* onshore tandem GAAA repeat-specific steady-state RNA is not detected in mononucleated blood cells from two healthy female donors (21 years old) or a male donor (33 years old) or from the THP-1 male cell line. RNA samples were either reverse (+) or mock (−) transcribed (RT) prior to PCR amplification across the *RP2* onshore tandem GAAA repeat-specific region (**A**) or the *GAPDH*-specific region (**B**). Corresponding samples of genomic DNA were used as positive controls for the PCR assays. The amplification products were separated via electrophoresis in an 8% acrylamide: bis-acrylamide gel and silver-stained for detection. Lane L50 shows a standard 50-bp ladder (Invitrogen); lane H_2_O is the negative PCR amplification control. The range of the *RP2* onshore tandem GAAA repeat-specific DNA amplimer is 350 to 391-bp. The *GAPDH*-specific DNA amplimers are as follows: 130-bp for *GAPDHP63* (6∶80663360-80663489) and *GAPDHP1* (X:39647022-39647151) and 220-bp for *GAPDH* (12∶6646089-6646308). The processed (mature) *GAPDH*-specific cDNA-derived product is 130- bp.

We performed reverse transcription-PCR experiments on total RNA from peripheral blood (normal women and men) and from the FANTOM-DB [33] human acute monocytic leukemia THP-1 reference cell line and found no detectable GAAA repeat-specific steady-state RNA (Figure 2). *In silico* PCR analyses using public RNA-Seq expression databases revealed no significant transcription activity across (or within) the *RP2* onshore tandem GAAA repeat locus in many different cell types and lines (Figure S4). However, the evidence does support the prediction of long RNA-Seq junctions based on ENCODE/CSHL, pooled from GM12878 whole-cell polyA (hg19 coordinates chrX:46545885-46727348). These long RNA-Seq junctions encompass multiple genes.

### Allelic distribution for the *RP2* onshore tandem GAAA repeat

**Figure 3:**
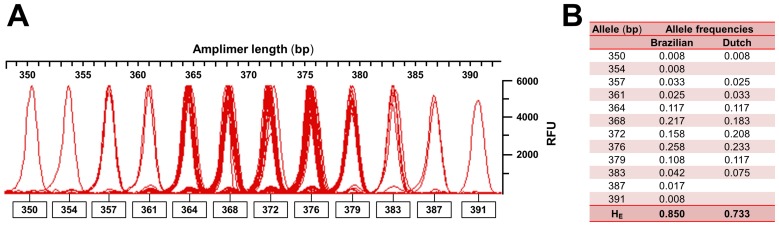
Allelic distribution of the *RP2* onshore tandem GAAA repeat. (**A**) Electropherogram of alleles observed in 60 unrelated Brazilian females genotyped via quantitative fluorescent PCR. The intensity of the red line tracing is related to the allele frequency. Smaller peaks preceding the designated allele peaks represent Taq polymerase stutter products corresponding to a mean of 2.6% of the amount of the true allele. In contrast, the mean stuttering for the *AR* disease-linked CAG repeat was 17.6% (not shown). Allele names are the lengths in base pairs of each fluorescence peak and the intensity of each peak is in relative fluorescence units (RFU). The *RP2* onshore tandem GAAA repeat locus exhibited an allelic span (the difference in length between the longest and the shortest allele per locus) of 41-bp in this population subset. (**B**) *RP2* onshore tandem GAAA repeat-containing allele frequencies and heterozygosity (H_E_) rates observed in the population subsets consisting of Brazilian and Dutch women.

The *RP2* GAAA onshore repeat-containing locus encompasses the reference upstream gene deletion/insertion variations rs6151299, rs373239539, rs201864594, rs201168201 and rs71950018. No validation had been reported for these variants (dbSNP build 138). We employed both the *RP2* onshore tandem GAAA repeat and the *AR* disease-linked, exonic CAG repeat in developing a biplex 5^me^CpG-based quantitative fluorescent PCR surrogate assay of human X-chromosome activity. For the determination of heterozygosity rates and allele frequencies, we genotyped two population subsets of sixty healthy unrelated women from Brazil and the Netherlands. For the *RP2* onshore tandem GAAA repeat, we observed up to twelve alleles with virtually no stuttering (Figure 3) in either subset. In the Brazilian subset, the heterozygosity rate for the *RP2* onshore tandem GAAA repeat was 0.85, matching that of the *AR* disease-linked CAG repeat (Figure S5). For the Dutch subset, the rate was 0.73, which was lower than that observed for the *AR* marker (0.87) (Figure S6). When the two subsets were pooled, the combined informativeness (e.g., at least one informative marker) of the *RP2*/*AR* biplex assay was 0.97.

### Methylation statuses of CpG sites near the human *RP2* onshore tandem GAAA repeat

**Figure 4:**
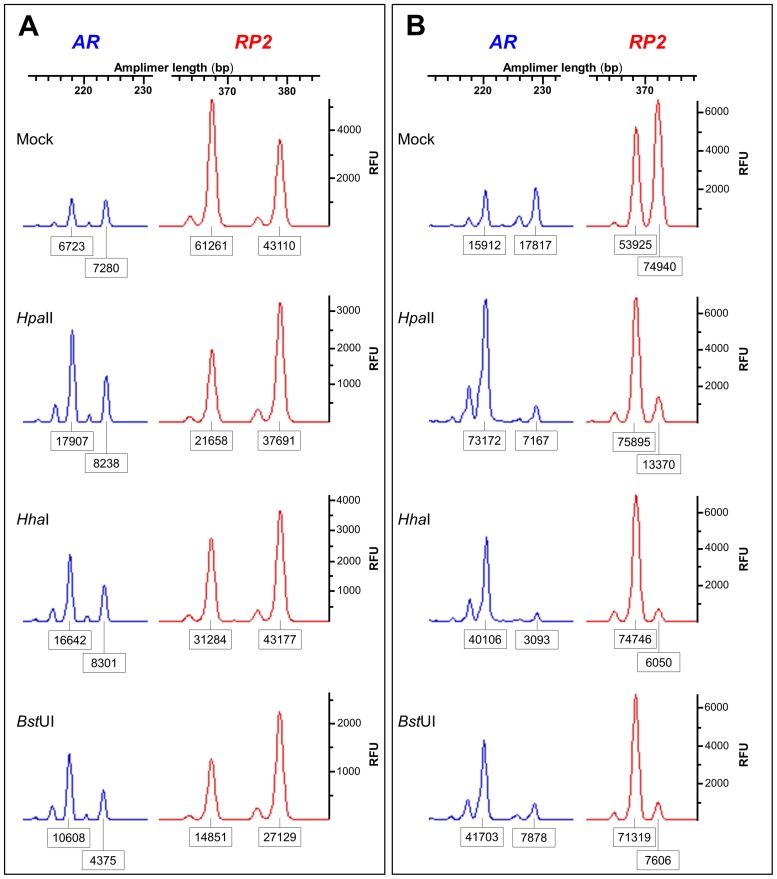
Methylation statuses at CpG sites near the *RP2* onshore tandem GAAA repeat. Random (**A**) and non-random (**B**) X-inactivation patterns generated for different CpG-containing 5^me^CpG-sensitive restriction endonuclease sites obtained using the 5^me^CpG-based PCR *RP2*/*AR* biplex assay across the restriction sites. Electropherograms of alleles observed in either undigested genomic DNA or DNA digested with *Hpa*II, *Hha*I or *Bst*UI from females genotyped via quantitative fluorescent PCR are shown. The boxed numbers correspond to the areas under the allele peaks and the intensity of each peak is in relative fluorescence units (RFU).

Each *RP2* onshore tandem GAAA repeat-containing allele comprises eight CpG sites, corresponding to five 5^me^CpG-sensitive restriction endonucleases (*Aci*I, *Bst*UI, *Fau*I, *Hha*I and *Hpa*II) and is therefore liable to multipoint 5^me^CpG interrogation. We used *Hpa*II, *Bst*UI and *Hha*I in XCI experiments, applying the 5^me^CpG-based PCR assay targeting the polymorphic repeat. The random (Figure 4A) and non-random (Figure 4B) patterns of X-inactivation obtained using these restriction enzymes were similar. We note, however, that the Xa/Xi lyonization ratios obtained using the *Hha*I and *Bst*UI enzymes were not always highly corresponding. This result may be related to the fact that in this particular target sequence the *Hha*I site overlaps the CpG within the *Bst*UI site and that overlapping CpG sites may block or impair cleavage if methylated (New England Biolabs usage guidelines). Therefore, this is a case where the overlapping CpG methylation cannot be predicted accurately.

### *RP2* and *AR* repeat-based methylation results are concordant

**Figure 5:**
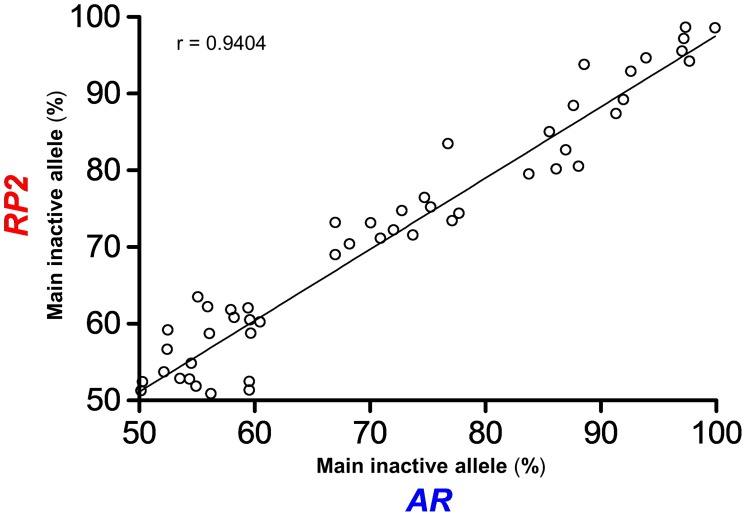
*RP2* and *AR* repeat-based methylation results are highly concordant. Scatterplot visual assessment of the strength of association between the percentages of the main inactive allele referred by the methylation statuses at the *RP2* onshore tandem GAAA repeat (*y-*axis) and the *AR* CAG repeat (*x*-axis) loci. The methylation statuses are highly concordant (Spearman r = 0.9404, CI95% = 0.8950 to 0.9665; *p*<0.0001) across varying degrees of random (50–80%) and non-random (>80%) XCI. The regression line superimposed on the plot provides the best-fitting straight line for the scattered data.

To correlate random and non-random X-inactivation patterns from the *RP2* onshore GAAA and *AR* CAG repeats, we genotyped a third subset of fifty unrelated women from Brazil and Argentina (Figure S7) and analyzed the CpG methylation statuses within the *Hpa*II sites. These women had known *AR* CAG repeat 5^me^CpG allele-specific profiles and, hence, known XCI ratios. The patterns of X-inactivation obtained using the *RP2*/*AR* repeat biplex assay were highly concordant (Spearman r = 0.9404; *p*<0.0001) (Figure 5).

**Figure 6:**
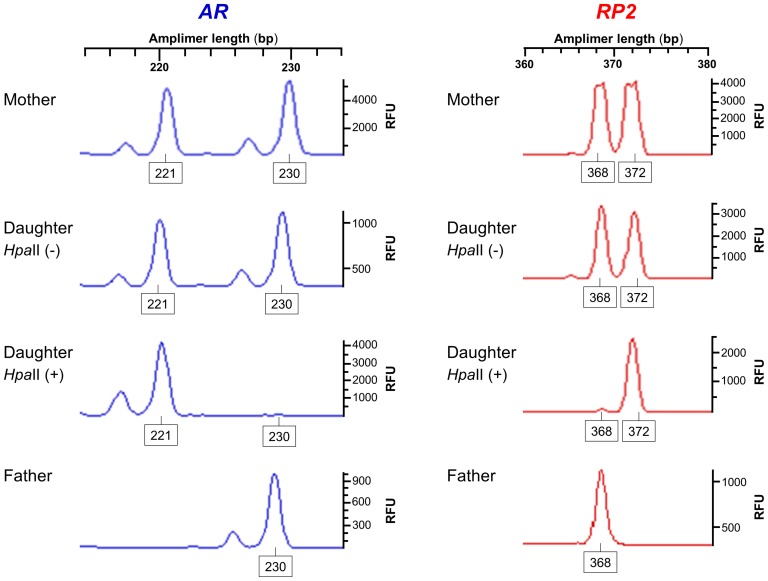
*AR* CAG and the *RP2* GAAA polymorphisms refer to the same X-chromosomes. Segregation analysis of either *AR* or *RP2* alleles distinguishes the maternal origin of the preferentially skewed Xi present in the daughter. Xi is identified based on the 230-bp *AR* allele and the 368-bp *RP2* allele. The allele names are the lengths in base pairs of each fluorescence peak and the intensity of each peak is in relative fluorescence units (RFU). Note that the magnitude of stuttering at the *RP2* onshore tandem GAAA repeat is minimal, in contrast with that at the *AR* CAG repeat.

To address the question of whether the *RP2* GAAA-containing alleles are located on the same Xa/Xi chromosomes identified based on the *AR* CAG-containing repeat, we determined the parent-of-origin of Xa and Xi in a nuclear family in which the normal daughter exhibited extremely skewed XCI in peripheral blood leukocytes (Figure 6). The segregation analysis demonstrated that the *AR* CAG and the *RP2* GAAA polymorphisms refer to the same X-chromosome based on correctly identifying the maternal origin (^M^X) of the preferential Xi in this nuclear family.

**Figure 7:**
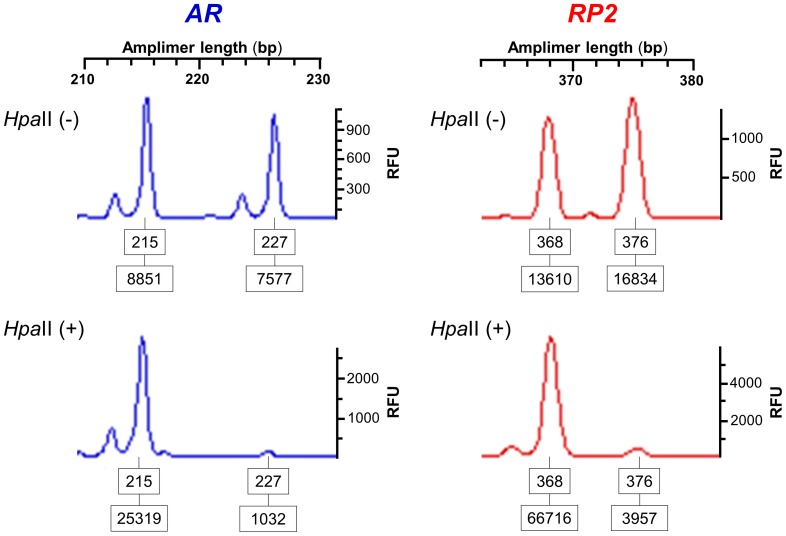
Hemophilia A occurs due to highly skewed XCI. Electropherograms of alleles obtained using the 5^me^CpG-based *RP2*/*AR* repeat biplex PCR assay across the *Hpa*II restriction site in a heterozygote female carrier of a one-base insertion, frameshift mutation in factor VIII domain B. The female is a hemophiliac due to highly skewed inactivation of the unaffected X-chromosome, represented by the *AR* 215-bp and *RP2* 368-bp alleles. The *RP2* and *AR* repeat-based 5^me^CpG readouts refer to the skewed X-inactivation state. The *F8* mutation was screened through conformational sensitive gel electrophoresis [19] and direct sequencing. Allele names (upper boxed numbers) are the lengths in base pairs of each fluorescence peak and the intensity of each peak is in relative fluorescence units (RFU). The lower boxed numbers correspond to the areas under the allele peaks.

To demonstrate the power of the *RP2* onshore tandem GAAA repeat in discriminating Xa from Xi in heterozygote carriers of an X-linked recessive defect that manifests through non-random XCI, we genotyped four confirmed heterozygous women affected by severe hemophilia A. Two of these individuals are conventional, non-symptomatic carriers who tested positive for *F8* intron 22 inversions via inverse shifting-PCR [34] and for random XCI based on the *AR* disease-linked CAG repeat assay; the other two are heterozygous carriers of missense and frameshift mutations in factor VIII domains A1 and B, respectively, and they present with symptoms of hemophilia A through non-random XCI. Again, the XCI patterns associated with the *RP2* onshore tandem GAAA repeat were highly concordant with those of the *AR* disease-linked CAG repeat, as exemplified in Figure 7 for a heterozygous female, hemophiliac due to highly skewed inactivation of the unaffected X-chromosome.

### The *RP2* onshore tandem GAAA repeat locus is conserved in nonhuman primates

**Figure 8:**
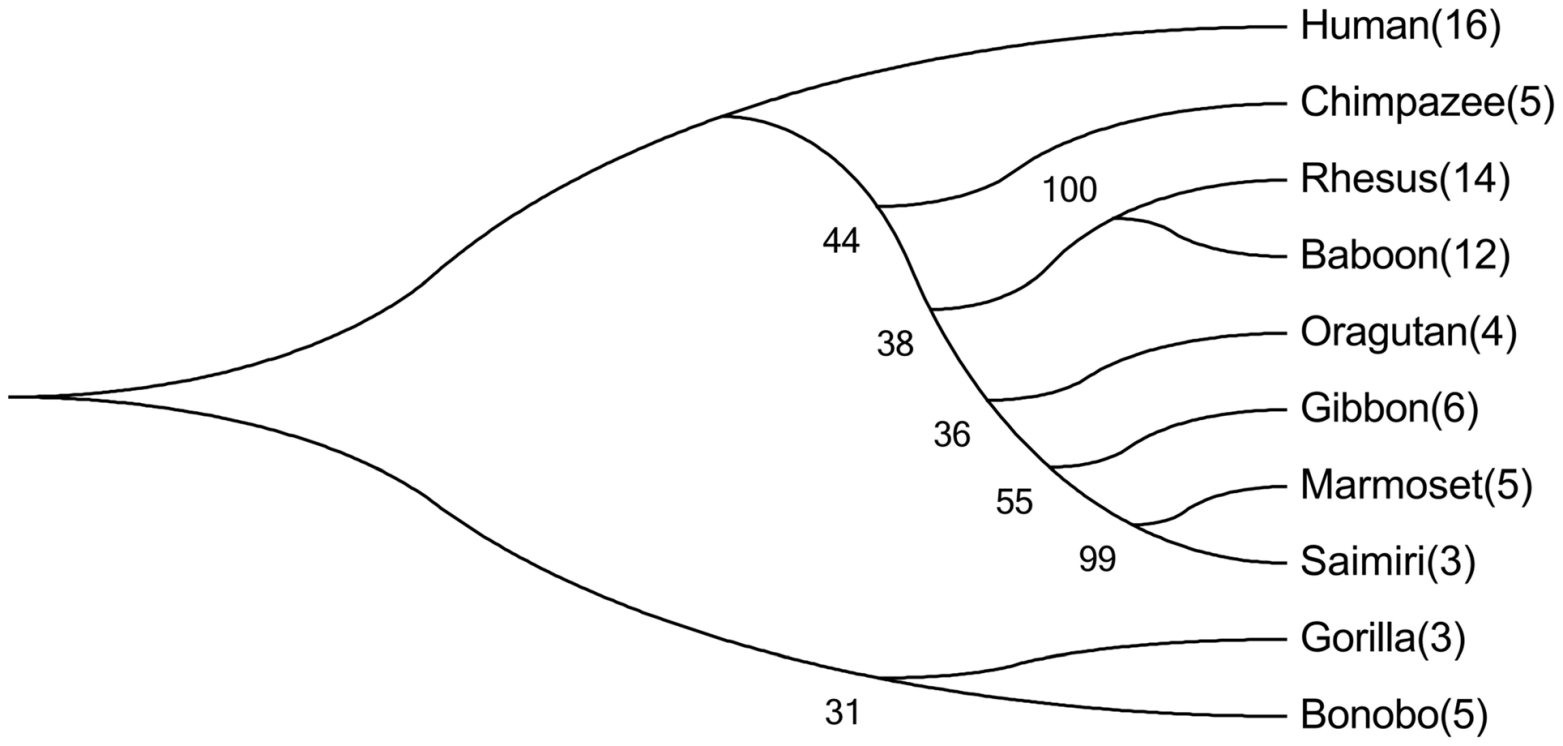
Molecular phylogenetic analysis using the maximum likelihood method. The evolutionary history was inferred using the maximum likelihood method based on the Tamura-Nei model [50]. The bootstrap consensus tree inferred from 1,000 replicates [51] is taken to represent the evolutionary history of the analyzed taxa [51]. The percentages of replicate trees in which the associated taxa clustered together in the bootstrap test (1,000 replicates) are shown next to the branches [51]. Initial tree(s) for the heuristic search were obtained automatically by applying the maximum parsimony method. The analysis involved 10 nucleotide sequences. The codon positions included were 1^st^ + 2^nd^ + 3^rd^+ Noncoding. In total, there were 410 positions in the final dataset. Evolutionary analyses were conducted in MEGA5 [25]. The numbers in parentheses correspond to the lengths of the uninterrupted tandem arrays in GAAA repeat units.

Although the *RP2* gene is conserved in mammals (data not shown), the *RP2* onshore tandem GAAA repeat locus is restricted to primates, as judged based on comparative *in silico* analyses using genomic reference sequences from public databases (Figure S8). This observation indicates that the insertion of the GAAA repeat element was a very recent event. The number of uninterrupted (perfect tandem array) GAAA repeat units varied from 3 (squirrel monkey) to 16 (humans) (Table S2 and Figure S9). We used the human *RP2* GAAA onshore repeat amplimer reference sequence, without masking the repeat region, to computationally search public data for homologs in primates, and we conducted evolutionary analyses with unmasked, masked or exclusion of repeat regions to construct a phylogenic tree (Figure 8). We found no evidence of a linear increase in the number of uninterrupted GAAA repeat units proportional to the time of divergence between nonhuman primates and humans.

### The *RP2* onshore tandem GAAA repeat is polymorphic in marmosets

**Figure 9:**
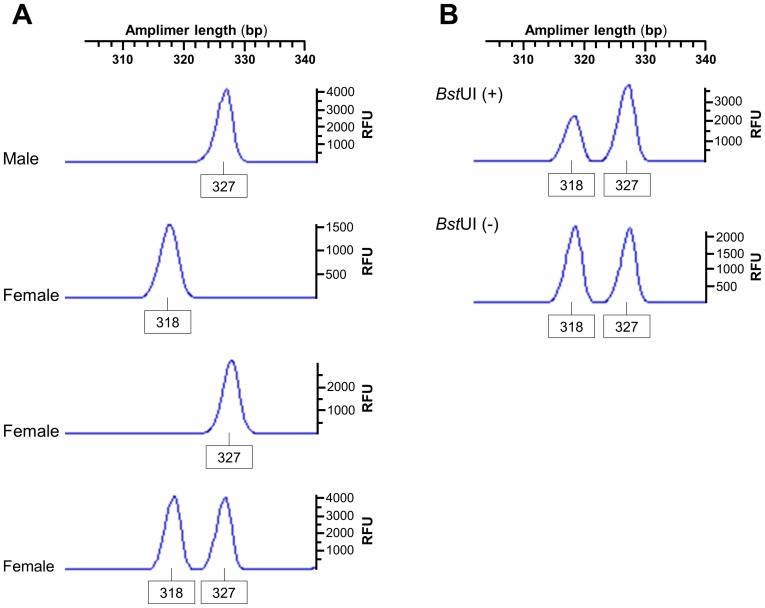
The *RP2* onshore tandem GAAA repeat is polymorphic in marmosets. Electropherograms of alleles observed in marmosets genotyped via quantitative fluorescent PCR. (**A**) Representative allele profiles from males, which exhibited only the major allele, and female animals with three distinct genotypes (homozygotes for either the minor or major allele or heterozygotes) are shown. (**B**) Representative random XCI pattern observed at the 5^me^CpG-sensitive *Bst*UI recognition site within the *RP2* GAAA-containing amplimer, with an Xa/Xi ratio of approximately 65%. The allele names are the lengths in base pairs of each fluorescence peak and the intensity of each peak is in relative fluorescence units (RFU).

We hypothesized that the *RP2* onshore tandem GAAA repeat locus may be useful in XCI studies in nonhuman primate species in which the *AR* CAG-repeat locus is not polymorphic [16]. We therefore tested this possibility in the naturally occurring, pervasive hematopoietic chimeric New World monkey marmoset (*Callitrichidae*) [35]. We observed only two alleles (318-bp and 327-bp) in 22 different animals (Figure 9A). All the males were monoallelic (hemizygous). The heterozygosity rate in females was 0.35. The *RP2* GAAA repeat-containing amplimer, as validated via *in silico* PCR, comprises five CpG sites, the methylation statuses of which can be determined with the restriction enzymes *Aci*I, *Bst*UI and *Fau*I. Here, we analyzed the 5^me^CpG-sensitive *Bst*UI recognition site (Figure 9B). For all heterozygote female marmosets tested, the pattern of methylation at the CpG site linked to the GAAA repeat of interest was random, with Xa/Xi ratios varying from 38 to 65%. Different tissues (blood, muscle, liver, brain and skin) from the same animal also yielded random, yet varying, Xa/Xi ratios (data not shown).

## Discussion

Notwithstanding the remarkable advances in understanding human genome structural variation and rapidly evolving technologies, the *AR* disease-linked CAG repeat-based HUMARA assay has remained the mainstay of XCI diagnosis in the two decades since it was reported [12]. Despite the elevated heterozygosity observed worldwide, there are important drawbacks to genotyping with exonic rather than neutral repeats. CAG repeat-associated non-ATG translation (RAN translation) can occur across human genes, and CAG repeat expansions in transcripts without an ATG result in the accumulation of toxic homopolymeric proteins in all three reading frames [36]. There is also evidence of bidirectional transcription of triplet repeat disease genes [37]. Moreover, PCR genotyping involving trinucleotide repeats is prone to template errors due to *in vitro* replication slippage by Taq polymerase [38], resulting in unwanted n-3 stutter products consisting of multiples of the true template alleles [39] to varying magnitudes, in a repeat sequence-dependent manner. Although several dinucleotide repeat loci have been proposed as supplements or alternatives to the *AR* disease-linked CAG repeat assay [40]–[42], the greater magnitude of n-2 stutter products is an unfortunate shortcoming, which can considerably influence the results and confound the analysis, as discrepancies in Xa/Xi ratios relating to the *AR* disease-linked CAG repeat assay have been reported [41], [42].

In contrast with the *AR* disease-linked CAG repeat (≥38 CAG repeat units are linked to KD [13]), the novel *RP2* onshore tandem GAAA repeat is endowed with neutral features. This observation suggests that expansions of the *RP2* onshore tandem GAAA repeat will not produce toxic RNAs that might otherwise influence cell viability, disease penetrance and pathological severity [43].

Data from a recent methylome study showed that the amplimer encompassing the human *RP2* onshore GAAA repeat spans eight CpG sites that are differentially hypomethylated in a tissue**-**dependent manner [44]. The same configuration occurs for the *AR* amplimer, but the levels of methylation are higher because the CpG sites are in the gene body. The observation that the Xa/Xi ratios inferred by determining the methylation statuses of CpG sites near the human *RP2* GAAA onshore repeat are highly concordant with the patterns of X-inactivation inferred from the HUMARA assay assuages the concerns related to typing the novel extragenic *RP2* onshore tandem GAAA repeat in XCI studies. We also showed that the extragenic *RP2* onshore tandem GAAA repeats and the neighboring CpG methylation statuses refer to exactly the same parental chromosomes identified based on the *AR* CAG repeat. Furthermore, it is known that the transcriptional XCI patterns generated by pyrosequencing correlate excellently (Pearson r^2^ = 0.96) with the XCI ratios reported using the HUMARA assay [45]. Thus, we feel confident that the analysis using the methylation statuses surrounding the *RP2* onshore tandem GAAA repeat will be as accurate as those obtained using the *AR* CAG marker in discriminating Xa from Xi chromosomes in other tissues and population subsets.

Evolutionary analyses of the *RP2* onshore tandem GAAA repeat locus indicated that the tandem arrangement is well conserved in nonhuman primates. Although there is a trend of directional expansion of the repeat, we see no evidence for a linear continuous increase in the length of a perfect tandem array proportional to the time since divergence from the last common ancestor. This observation contrasts with findings related to the *AR* CAG exonic repeat, for which a linear increase in triplet repeat length proportional to the time since divergence has been reported twice [16], [46].

Because of its proximity to known *RP2*+1 transcriptional start sites and its polymorphic nature, the *RP2* onshore tandem GAAA repeat could be regarded as a core promoter STR and may be a source of variation across species [47]. Whether the GAAA repeat expansion plays a role in *RP2* gene expression leading to inter**-**individual variation is currently unknown.

The *RP2* onshore tandem GAAA repeat was less polymorphic in marmosets than in humans, with only 2 alleles being observed in 22 animals. The marmoset reference genomic sequence bears only five uninterrupted GAAA repeat units, represented by the observed major (e.g., the most frequent and oldest) 327-bp allele. This result suggests that in marmosets, the *RP2*-extragenic GAAA locus may correspond to stable, fixed (GAAA)_5>3_ deletion/insertion biallelic variation. Given that the highest possible heterozygosity rate for any biallelic system is 50%, the observed heterozygosity rate of 35% is highly significant. Alternatively, this result can be explained by reduced genetic diversity due to a limited number of founder animals in the studied primate colony, as reported for the CAG *AR* repeat in nonhuman-primates [16] and/or functional restriction of the ability of the repeat to expand in these species. We are currently addressing the latter possibility. Nevertheless, the observed polymorphism in marmosets enabled us to develop a molecular genotyping assay to study XCI in a small nonhuman primate experimental model in which the *AR* disease-linked CAG repeat locus is known to be monomorphic [16].

## Conclusions

The superior efficacy of the 5^me^CpG-based *RP2*/*AR* repeat biplex assay in differentiating the parental origins of Xa and Xi chromosomes in approximately 97% of human females constitutes a notable advance in the field of XCI, and this assay excels at determining the 5^me^CpG statuses of alleles on the Xp (*RP2*) and Xq (*AR*) chromosome arms in a single reaction. The *RP2* onshore tandem GAAA repeat will facilitate studies on the variable phenotypic expression of dominant and recessive X-linked diseases (e.g., Rett syndrome, hemophilia A and B, mental disability), epigenetic changes in twins, the physiology of aging hematopoiesis, the pathogenesis of age-related hematopoietic malignancies and the clonality of cancers in human and nonhuman primates [48].

## Supporting Information

Figure S1 **Computational validation of a polymorphism at the** ***RP2*** **onshore tandem GAAA repeat locus in reference genome sequences.**
(DOC)

Figure S2 **Physical positions of known transcription start sites, transcription factor binding sites and predicted microRNA precursors relative to the** ***RP2*** **onshore tandem GAAA repeat locus.**
(DOC)

Figure S3 **The** ***RP2*** **onshore tandem GAAA repeat locus does not overlap with** ***RP2*** **cDNAs or cap analysis gene expression promoters (CAGE).**
(DOC)

Figure S4 **RNA-Seq evidence across the human** ***RP2*** **onshore tandem GAAA repeat locus.**
(DOC)

Figure S5 **Distribution of distinct genotypes for the** ***RP2*** **onshore tandem GAAA repeat (A) and** ***AR*** **tandem CAG repeat (B) loci in the first population subset (n = 60 Brazilian females).**
(DOC)

Figure S6 **Distribution of distinct genotypes for the** ***RP2*** **onshore tandem GAAA repeat (A) and** ***AR*** **tandem CAG repeat (B) loci in the first population subset (n = 60 Dutch females).**
(DOC)

Figure S7 **Genotypes for the** ***RP2*** **onshore tandem GAAA repeat (A) and** ***AR*** **tandem CAG repeat (B) loci in the third population subset (n = 46 Brazilian females and n = 4 Argentinean females), consisting of women with known** ***AR*** **tandem CAG repeat 5^me^C allele-specific profiles and, hence, known XCI ratios.**
(DOC)

Figure S8 **The** ***RP2*** **onshore tandem GAAA repeat locus is conserved in primates.**
(DOC)

Figure S9 **Multiple sequence alignment of the** ***RP2*** **onshore tandem GAAA repeat locus reveals high conservation in primates.**
(DOC)

Table S1 **PCR primer sequences used in this study.**
(DOC)

Table S2 **Structure of the** ***RP2*** **onshore tandem GAAA repeat region in primates.**
(DOC)
