# Supplementary material for: 5meCpG Epigenetic Marks Neighboring a Primate-Conserved Core Promoter Short Tandem Repeat Indicate X-Chromosome Inactivation
Source: PLoS One. 2014 Jul 31;9(7):e103714. doi: 10.1371/journal.pone.0103714 (PMC4117532; doi:10.1371/journal.pone.0103714)
Supplement: Figure S1 — Computational validation of a polymorphism at the RP2 onshore tandem GAAA repeat locus in reference genome sequences. (DOC) [file pone.0103714.s001.doc]

**Figure S1**. **Computational validation of a polymorphism at the *RP2* onshore tandem GAAA repeat locus in reference genome sequences**. Clustal W [1] multiple sequence alignment of the region containing the *RP2* onshore tandem GAAA repeat in three reference X chromosome sequences available from NCBI with the accession numbers NC_000023 (reference assembly), AC_000066 (alternate assembly, based on Celera assembly) and AC_000155 (alternate assembly, based on HuRef). The primer sequences used for PCR genotyping are highlighted in red and pink; the GAAA repeat polymorphism is highlighted in yellow.

ref|NC_000023.10|_46695746-466 TGACATAGCGAGACCCTGTGAAAGAAAAAGAAAGAAAGAAAGAAAGAAAG

ref|AC_000066.1|_50887596-5088 TGACATAGCGAGACCCTGTGAAAGAAAAAGAAAGAAAGAAAGAAAGAAAG

ref|AC_000155.1|_44407317-4440 NNNNNNNNNNNNNNNNTGTGAAAGAAAAAGAAAGAAAGAAAGAAAGAAAG

**********************************

ref|NC_000023.10|_46695746-466 AAAGAAAGAAAGAAAGAAAGAAAGAAAGAAAGAAAGAAAGAGCACAGAAG

ref|AC_000066.1|_50887596-5088 AAAGAAAGAAAGAAAGAAAGAAAGAAAGAAAGAAA----GAGCACAGAAG

ref|AC_000155.1|_44407317-4440 AAAGAAAGAAAGAAAGAAAGAAAGAAAGAAAGAAA----GAGCACAGAAG

*********************************** ***********

ref|NC_000023.10|_46695746-466 AGGATTGGGAGGTTATGGGGTACAATTCTTGAGGGGGTGACCCTGGCCAG

ref|AC_000066.1|_50887596-5088 AGGATTGGGAGGTTATGGGGTACAATTCTTGAGGGGGTGACCCTGGCCAG

ref|AC_000155.1|_44407317-4440 AGGATTGGGAGGTTATGGGGTACAATTCTTGAGGGGGTGACCCTGGCCAG

**************************************************

ref|NC_000023.10|_46695746-466 GCGATGAGAGGGCGGTAGGAAGGGTGATGAGGGGAGAAGAGGATTTAGAA

ref|AC_000066.1|_50887596-5088 GCGATGAGAGGGCGGTAGGAAGGGTGATGAGGGGAGAAGAGGATTTAGAA

ref|AC_000155.1|_44407317-4440 GCGATGAGAGGGCGGTAGGAAGGGTGATGAGGGGAGAAGAGGATTTAGAA

**************************************************

ref|NC_000023.10|_46695746-466 ATACAAATTTCAGGGTCTTCTTGTGTCAGCGGGAATTTCTGTCCCTCACA

ref|AC_000066.1|_50887596-5088 ATACAAATTTCAGGGTCTTCTTGTGTCAGCGGGAATTTCTGTCCCTCACA

ref|AC_000155.1|_44407317-4440 ATACAAATTTCAGGGTCTTCTTGTGTCAGCGGGAATTTCTGTCCCTCACA

**************************************************

ref|NC_000023.10|_46695746-466 ACTTTCATCATAAGATAAATCTAATGTTCAACTAGAGATCTCTCCCGCGC

ref|AC_000066.1|_50887596-5088 ACTTTCATCATAAGATAAATCTAATGTTCAACTAGAGATCTCTCCCGCGC

ref|AC_000155.1|_44407317-4440 ACTTTCATCATAAGATAAATCTAATGTTCAACTAGAGATCTCTCCCGCGC

**************************************************

ref|NC_000023.10|_46695746-466 CTTGAACTTGCAAATTTATGAATCAGGGGCAAAAAAAACCCGGATACCGA

ref|AC_000066.1|_50887596-5088 CTTGAACTTGCAAATTTATGAATCAGGGGCAAAAAAAACCCGGATACCGA

ref|AC_000155.1|_44407317-4440 CTTGAACTTGCAAATTTATGAATCAGGGGCAAAAAAAACCCGGATACCGA

**************************************************

ref|NC_000023.10|_46695746-466 GCCTGGCCTCCCACCAGCTAGAGAACCCACCAa

ref|AC_000066.1|_50887596-5088 GCCTGGCCTCCCACCAGCTAGAGAACCCACCAa

ref|AC_000155.1|_44407317-4440 GCCTGGCCTCCCACCAGCTAGAGAACCCACCAa

*********************************

**References**

1. Thompson JD, Higgins DG, Gibson TJ (1994) CLUSTAL W: improving the sensitivity of progressive multiple sequence alignment through sequence weighting, position-specific gap penalties and weight matrix choice. Nucleic Acids Res 22: 4673-4680.
