# Supplementary material for: 5meCpG Epigenetic Marks Neighboring a Primate-Conserved Core Promoter Short Tandem Repeat Indicate X-Chromosome Inactivation
Source: PLoS One. 2014 Jul 31;9(7):e103714. doi: 10.1371/journal.pone.0103714 (PMC4117532; doi:10.1371/journal.pone.0103714)
Supplement: Figure S2 — Physical positions of known transcription start sites, transcription factor binding sites and predicted microRNA precursors relative to the RP2 onshore tandem GAAA repeat locus. (DOC) [file pone.0103714.s002.doc]

**Figure S2**. **Physical positions of known transcription start sites**, **transcription factor binding sites and predicted microRNA precursors relative to the *RP2* onshore tandem GAAA repeat locus**. Reference sequence for the 5-kb fragment upstream of known *RP2* transcription start sites (highlighted in turquoise and bold letters), the distribution of transcription factor binding sites (TFBS; highlighted in grey) and predicted microRNA precursors (highlighted in green) are shown. The locations of the forward and reverse primers used for genotyping the *RP2* onshore tandem GAAA repeat are highlighted in red and pink, respectively. The tandem GAAA repeat is highlighted in yellow. The positions of the microRNA precursors was determined using the [CID-miRNA](http://mirna.jnu.ac.in/cidmirna/) browser [1]; the positions of the Transcriptional Start Sites (TSS) and TFBS were assessed using the [TRED](http://rulai.cshl.edu/TRED) [2], [DBTSS](http://dbtss.hgc.jp/) [3], [FANTOM](http://fantom.gsc.riken.jp/4/gev/gbrowse/hg18/) [4] and [SwissRegulon](http://www.swissregulon.unibas.ch/cgi-bin/regulon?page=swissregulon) [5] public web portals. Within the sequence map, the following MotEvo CAGE [6] and Swissregulon [5] TFBS are shown: GFI1B-97789 (GGCTTTGATTGG), NFYA,B,C-154045 (GCTTTGATTGGCTCAA), GFI1-134300 (CTTTGATTGG), SOX2-220379 (CTTTGAT), PBX1-162809 (TTTGATTGGCTC), NFYA,B,C-154046 (GCGTTCATTGGCTGCG), KLF4-135599 (AAGGGGAAGG), TBX4,5-96119 (AGGGGTGCTGGG), ZNF384.p1-542867 (CAAAAAAAA), XCPE1[core].pl-510857 (GGGCGGGGCC), FOXM1.p1-122818 (GCTTTGATT), SOX17.p1-410238 (TTCATTGGC), TBX4,5.p1-468439 (AGGGGTGCTGGG), KLF4.p1-218948 (AAGGGGAAGG) and PRDM1.p1-381741 (AAAAAGGGGAAGGG).

>gi|224589822:46691347-46696395 Homo sapiens chromosome X, GRCh37.p5 Primary Assembly

GAAGCCTTGCAGCTTCCACGTTGGTCTCATGGAACACTCACTCTTGGAGCCCTGAGCATGATGTATGGAGTTCAAATACCCTAAAGCCACCTTGCAGGAGAGACTGGGTGGAGAGGCTCTGAGATTTCATGTAGAATGATTCTTGGCCAGCACCCCAGCCATTTGAGTCATCCCAGCTGAGGCTCCAGATGTACTGTGGAACAGAATTGAGTTGTCCCCACCAAGGTTCATGAGTAAAAGAAATATTAAGTTATTATTGCTTTAAGCGATTACGTTTCAGGTGGTTTGTTACTCAGCAATAGATGACTGAAACACTCCTCTTTCACTTGATGTGAAGTATTTCCTTAGGTGAGTGCAGAGTGAGCAAGGATGTGTCAGGTCAACTCAAACATAGGGAATTTTCCAGCCATCGTTGCTGGGGTAATTGACTTATTCATGCTGTAGAGATACACTGCTTCAGGACTGTCTTTTTGTTGTTGTTGTTTTTGAGATGGGATCTCACTCTGTTGCCCAGGCTGGAGTGCAGTGGTGCGATCTTGGCTCACTACAGCCTCTGCCTCCCGGGTTCAAGCAATTCTCCCACCTCAGCCTTCTGAGTAGCCGGGATTACAGGGGCGCACCACCACGCCTGGCTAATTTTTGCATTTTTAGTATGTTTAGTATTTTCACCATGTTGACCAAGCTGGTCTCAAACTCCTGACCTCAAGTGATCCACCTGCCTCGGCCTCCCAAAGTGCTGGGATTACAGGTGTGAGCCACCGTGCCCGGCCAGGACTGTCTTCTGAGAAGTAACTATATCACTTCTGTGGTCAAGACTCAGGGGACCCAGAGCCATCTCTGGAGCTTGTGGCCAGGTGTAAACCCACAACTCAACATTTTAATATAGATGAATAGATACATCAAGCTGAACAGGAAGGGCAGCCTTCTATAGAAAAGAAATCACTAGAATATAAATAGCAGGAGGAGCACAAGCAGAACAAGCAACCAACTTCTGCCTGAACTCTTCAGAGTTAGCGTTCCCTTTTCAGGACCTGCTTCCCACCCATATCTGTAACACCCATGTTTGGAATTCGCCCCTGCAGCTGCAGCAATGGGACCAGGGGTGCATACCAACCTAGAGCCAATCTGTTATTGACTGGCCAGCAATGTATATGATTATCTCCCTTGAGAGTCTGAGACTGTAGCATGCCCTTGGACAGAAAGGCCATGAGGAATCAGGGTGGGATAGCTGTTGTCAACCATGTAAATGCTGATGAGTAAGTAGAGAGCTGGTTTGCAAAAATAAGTAGTGATGAGAATTCATGGGGACAGGTGAAAAAGAGATGGGGCAGAAAAGAAAGAAAGAATAAGAGAGAAAGAAAAAGGATGAGACAGCACTAGTATTAGCTCTGTTCATTAGCTTTCTAGTCCCCAGTTCTTAACATTTGATTTAATTTTCTTTTTGTAATTTTGTTTTTGGTCAAGAGTGATGGTAGGATGGGAATGAACAGAAAAGGGACTTTATCTTCACTTTTAAGGGTCCCTGAAAAGATCACTCTTGGATATAGGTTTTATTGTAGATTTGAGCTTTTCTCATTAATGAATACTTCAAAACAGTGAGAGTTTCAGAGGTTGTTTCTCTAGAATTAGTTTTGTTATATTGTTTGTTCATTAAGAGGAATAGCATCACTGTGGGACAGAGCTCCCATAGGCATCAGAAGGCCGAGGCAGATACTTATTTCAGATGAATTAGAGAGGAACAAACTTAAGGCAGGAGGAGGCACTGGGAAACACAGGATGTGGATTTTTCCAGTCCCTGCTCAGCCACTCTCATCGTGACCACAGATTCATCCCTTCACCTCTCTGGATCTCTGCCTCCTCTGCTACACTAAGTAGTGGTCATAGTTATATATTTATATCTGGTCACAGATGTTTTATAATCATAGACCATAGATTATAAATATAATAATAGATTATAAAATATCGAATGAGGGTCTTATCCAAAAACATCTTCACATAAAACTTTCATCTCACTCTGAAGGAGAAAAAGTGTGCACTGGGTAAAGACTCTATTGCCTAAGATCTGGAACTTAAATTTGCAGTAATTTCTAATACTTAAGCACATGTACTTTTCTTCCCGATTGTGCAATAAAGGACGGGAAGTAAAAAGGGGAACATTCTTTATGTTCAAATTAAGTCGGCCGGGCACTGTAGCTCACGCCTGTAATCCCAACATTTTGGGAGGCCGAGGTGGGTGGATCACCTGAGGTCAGGAGATCGAGACCAGCCTGGCCAACATGGTGAAACCCCGTCTCTACTAAAAATACAAAAATTAGCCAGGTGTGGTTTTGTGCGCCTGTAGCCCCAGCTACTCGGGAGGCTGAGGCAGGAGAATTGCTTGAACCTGGGAGGTGGAGGTTGCAGTGAGCTGAGATCATGTCATTGCACTCCAGCCTGGGTGATAAAGCGAGACTCTGTCTCAAAAAAAAAAAAAAAAAAAAAAGTCAGAGGCTCATTTAGGATACAAACTAGATGTGAATAAGTATGGGAAATTTAAAAGATAACCAAATTTGAACCTGAAGTGATCAGCATTTGATATCCTACTGAGATGTCAAAATACTGTTTCTCAGAGAAACTGACTTACCTTTCTTTTCTATTACATAGGCAATGCATGTGTATTGCAGTAAAATTCGACCCAAGTATAACAAATGTTTATATTTAGTGTATATATCTTTAAATATATTCTGACGAACCCTATGCCATTTGCTTAGAACAGATCTGATCGTTCATATGCAATGCTTATGTCCTCAACGAGGCTATAAACTCCATGAGGAGAACCCCTTTACCATAAATCCACATGCCTCCCACCCCCACAGTTTGGTCACTTTCTTTGGGAAGGGATGGGGGAAGTTTATAAATGCTTCCTTCACTAGCACCCATGCATGCATTCATTCATTTGACAAACGTTGTATTGAGCACCTAGTATGGACTTGGGCCAGGTGTAGGAGATTCAAAGGTACATCCCTCCCCTCAGCTTTCCTCCCCACTGCCTTTTTTTTTTTTTTTTTTTGAGACGGAGTCTTGCTCTGTCGCCCAGGCTGGAGTGGAGTGGCGCCATCTCGGCTCACTGCAACCTCCGCCTTCTGGGTTCAAGCTATTCTCCTGCCTCAGCCTCCCGAGTAGGTGGGACTACAGGCGCCCGCCACCATGCCCGGTTAATTTTTGTATTTTTAGTAGAGATGAGGTTTCACCATATTGGCCAGGCTGGTCTCGAACTCCTGACCTTGTGATCCGCCCGCCTTGGCTTCCCAAAGTGCTGGGATTACAGGCGTGAGCCACCGTGCCCGCCCCCCCCGGCTCTGGTTTTTTTTTTTTTTTTTTTTTTTAGACAGAGTCTCCCTCTGTCGCCCAGGCTGGAGTGCAGCGGCGCCATCTTGGCTCACTGCAACCTCCACCTCCTGGGTTCAAGCAATTCTCCTGCCTCAGCCTCCCGAGTAGCTGGGACTACAGGCACACGCCACTACGCCCGGCTAATTTTTGTATTTTTAGTAGAGACGGGGTTTCACCAGACGGGGTTTCACTGTGTTGAGCAGGATGGTCTCGATCTCCTGACCTCATGATCCTACCGCCTCGGCCTCCCAAAGTGCTGGGATGACAGCCGTGAGCCACCGCGCCCGGCCCTGCATGCCCTTTATGACATACCTTTTAGGCCAGGCGCGGTGGCTCATGCCTGTCATCCCAGCACTTTGGGAGGCCGAGGCGGGCAGATCACCTGAGGTCAGGAGATCGAGACCAGCCTGCCCAACATGGTGAAACCCCGTCTCTACTAAAAATACAAAAATTAGCCGGGCGTAGTGGCAGGCGCCTGCAGTCCCAGCTACTCTGGAGGCTGAGGCAGGATAATCACTTGAACCCGGGAGGCGGAGGTTGCAGTGAGCCGAGATGGCACTGCTGCACTCCAGCCTGGGCGACAAGAGCAAAAACTCCATCAAAAAAAAAAAAAAAAAAAAAAGATATACCTTTTTGATCTCAAGAAAGTCACAGTTCAAATAGGGAAACAATACACAATTAACTATTTTACTAAGTACTTGTGCAATTTTAGAGCCATATGTGAAAGTCTTAAGAAAACACAGAAGCAGCCTGGGGTGCGGTGGGTCCTGCCTGTAATCCCAGCACCTTGAGAGGCTGAGGCAGGATGATCGCTTGGGCCCAGGAGTTCAAGACCAGCCTGGGCGACATAGCAAGACCCCATTTCTACAAAAGTAAAAAAAAAAAAAAATAGGTGGGCATGGTGGTGCTTGCTTGTAGTTCCAGCTACTCAGGAAGGAGGCTGAGGCAGGAGGATCGCTTGAGCCCAGGAAGTCAAGGTTGCAAAGTTGCAAGGTTGCAGTGAGTTATGATTGTGCCACTGCAGCCTGAGTGACATAGCGAGACCCTGTGAAAGAAAAAGAAAGAAAGAAAGAAAGAAAGAAAGAAAGAAAGAAAGAAAGAAAGAAAGAAAGAAAGAAAGAGCACAGAAGAGGATTGGGAGGTTATGGGGTACAATTCTTGAGGGGGTGACCCTGGCCAGGCGATGAGAGGGCGGTAGGAAGGGTGATGAGGGGAGAAGAGGATTTAGAAATACAAATTTCAGGGTCTTCTTGTGTCAGCGGGAATTTCTGTCCCTCACAACTTTCATCATAAGATAAATCTAATGTTCAACTAGAGATCTCTCCCGCGCCTTGAACTTGCAAATTTATGAATCAGGGGCAAAAAAAACCCGGATACCGAGCCTGGCCTCCCACCAGCTAGAGAACCCACCAAGTTCCAGGAGGGGCGGGGCCGAGGGCGGTCCGTGCCACAGCTGTGGGCGGGGTTGTCCGTGCTTGCGCCAGAGCCGGCCCGCCCCGCCGCCAGGGGCTGTCGCAGGAGCCAGGCGTGGATTGGTATGTGGGTATGTCGGCCTCTCTTCATAGGCCGCGCAGGATTGCTCGAGAGGCTTTGATTGGCTCAACAGGCGCTGAGCGTTCATTGGCTGCGCT**G**GGTCGTCAGGGCGAC**G**AAAAAGGGGAAGGGGTGCTGGGCCTGGCGGG**C**

**References**

1. Tyagi S, Vaz C, Gupta V, Bhatia R, Maheshwari S, et al. (2008) CID-miRNA: a web server for prediction of novel miRNA precursors in human genome. Biochem Biophys Res Commun 372: 831-834.

2. Jiang C, Xuan Z, Zhao F, Zhang MQ (2007) TRED: a transcriptional regulatory element database, new entries and other development. Nucleic Acids Res 35: D137-140.

3. Yamashita R, Wakaguri H, Sugano S, Suzuki Y, Nakai K (2010) DBTSS provides a tissue specific dynamic view of Transcription Start Sites. Nucleic Acids Res 38: D98-104.

4. Kawaji H, Severin J, Lizio M, Forrest AR, van Nimwegen E, et al. (2011) Update of the FANTOM web resource: from mammalian transcriptional landscape to its dynamic regulation. Nucleic Acids Res 39: D856-860.

5. Pachkov M, Erb I, Molina N, van Nimwegen E (2007) SwissRegulon: a database of genome-wide annotations of regulatory sites. Nucleic Acids Res 35: D127-131.

6. Suzuki H, Forrest AR, van Nimwegen E, Daub CO, Balwierz PJ, et al. (2009) The transcriptional network that controls growth arrest and differentiation in a human myeloid leukemia cell line. Nat Genet 41: 553-562.
