# Supplementary material for: 5meCpG Epigenetic Marks Neighboring a Primate-Conserved Core Promoter Short Tandem Repeat Indicate X-Chromosome Inactivation
Source: PLoS One. 2014 Jul 31;9(7):e103714. doi: 10.1371/journal.pone.0103714 (PMC4117532; doi:10.1371/journal.pone.0103714)
Supplement: Figure S3 — The RP2 onshore tandem GAAA repeat locus does not overlap with RP2 cDNAs or cap analysis gene expression promoters (CAGE). (DOC) [file pone.0103714.s003.doc]

**Figure S3**. **The *RP2* onshore tandem GAAA repeat locus does not overlap with *RP2* cDNAs or cap analysis gene expression promoters** (**CAGE**). The positions of the *RP2* onshore tandem GAAA repeat locus and the landmark CpG island of the *RP2* promoter are indicated by the brown lines labeled **trf** and **CpG**, respectively. The locations of known CAGE promoters are indicated with brown arrows. The image was generated using the Human (hg18) [FANTOM](http://fantom.gsc.riken.jp/4/gev/gbrowse/hg18/) [1] genome viewer for THP-1 analysis [Release 2009/03/02] with the *RP2* onshore tandem GAAA repeat-containing region viewing coordinates chrX:46580500..46581600.


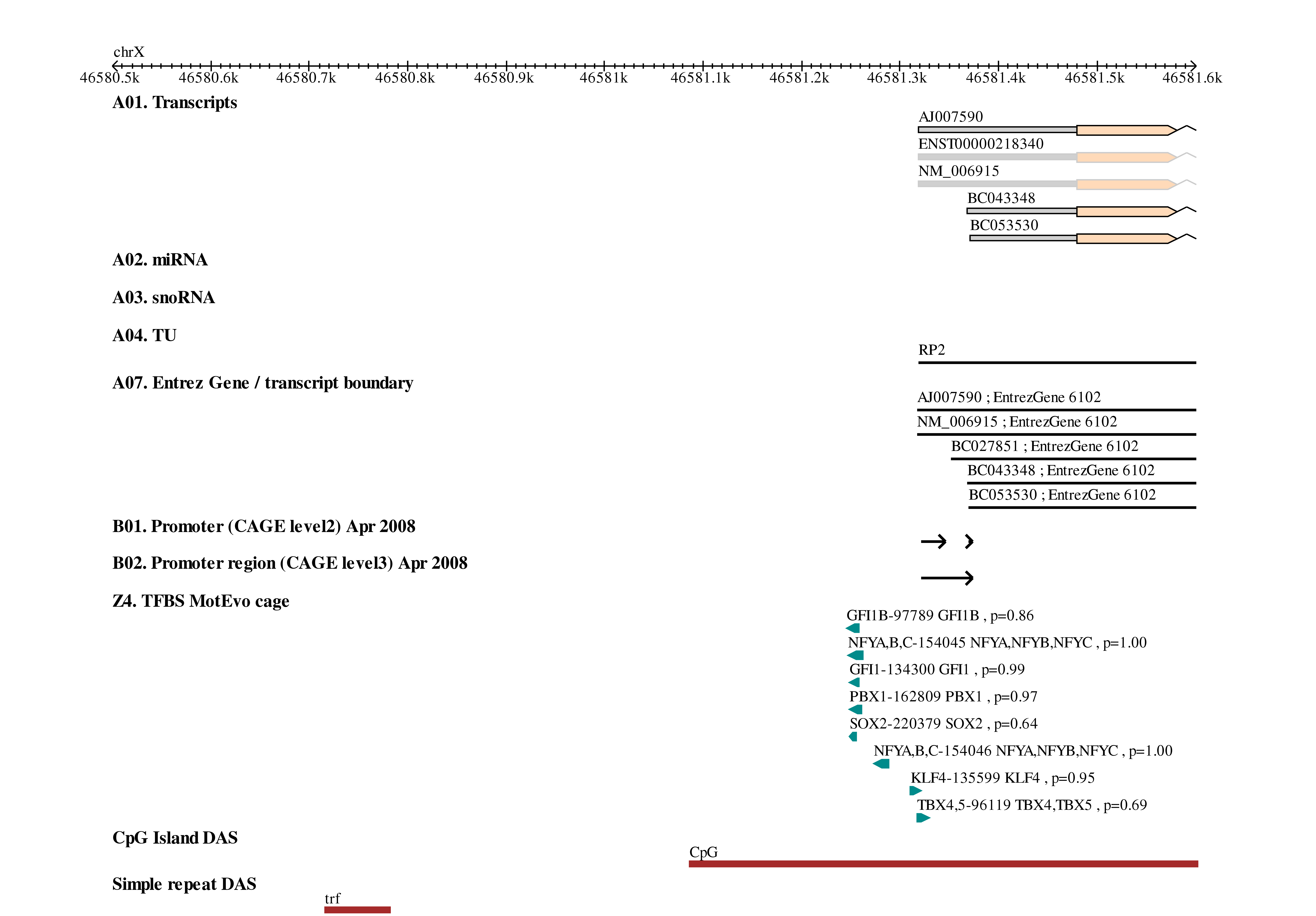


**References**

1. Kawaji H, Severin J, Lizio M, Forrest AR, van Nimwegen E, et al. (2011) Update of the FANTOM web resource: from mammalian transcriptional landscape to its dynamic regulation. Nucleic Acids Res 39: D856-860.
