# Supplementary material for: 5meCpG Epigenetic Marks Neighboring a Primate-Conserved Core Promoter Short Tandem Repeat Indicate X-Chromosome Inactivation
Source: PLoS One. 2014 Jul 31;9(7):e103714. doi: 10.1371/journal.pone.0103714 (PMC4117532; doi:10.1371/journal.pone.0103714)
Supplement: Figure S4 — RNA-Seq evidence across the human RP2 onshore tandem GAAA repeat locus. (DOC) [file pone.0103714.s004.doc]

**Figure S4**. **RNA-Seq evidence across the human *RP2* onshore tandem GAAA repeat locus**. A graphic display (available at the UCSC Genome Browser ([http://genome.ucsc.edu](http://genome.ucsc.edu/)) [1]) of the *in silico* PCR alignment of the 383-bp amplimer used in this study (hg19 genome assembly coordinates: chrX:46695746+46696128) is shown. The amplimer (upper track) is represented by solid blocks in which the primers (forward primer: 5' TGACATAGCGAGACCCTGTG 3'; reverse primer: 5' GTGGTGGGTTCTCTAGCTG 3') are located, with a double line indicating the sequence between them. The amplimer encompasses the *RP2* onshore tandem GAAA simple repeat locus, which was annotated using the RepeatMasker program (<http://www.repeatmasker.org/>). The following public tracks for RNA-Seq expression databases were used: CSHL Long RNA-Seq, ENCODE/Caltech, GIS RNA-Seq, HAIB RNA-Seq and SYDH RNA-Seq. The evidence is overwhelming in opposition to significant transcriptional activity across (or within) the *RP2* onshore tandem GAAA repeat locus in many different cell types and lines. However, the evidence does support long RNA-Seq junctions base on ENCODE/CSHL, pooled from GM12878 (B-lymphocyte, lymphoblastoid) whole-cell polyA RNA-Seq (hg19 coordinates chrX:46545885-46727348). These long RNA-Seq junctions encompass multiple genes.





**References**

1. Kent WJ, Sugnet CW, Furey TS, Roskin KM, Pringle TH, et al. (2002) The human genome browser at UCSC. Genome Res 12: 996-1006.
