# Supplementary material for: 5meCpG Epigenetic Marks Neighboring a Primate-Conserved Core Promoter Short Tandem Repeat Indicate X-Chromosome Inactivation
Source: PLoS One. 2014 Jul 31;9(7):e103714. doi: 10.1371/journal.pone.0103714 (PMC4117532; doi:10.1371/journal.pone.0103714)
Supplement: Figure S5 — Distribution of distinct genotypes for the RP2 onshore tandem GAAA repeat (A) and AR tandem CAG repeat (B) loci in the first population subset (n = 60 Brazilian females). (DOC) [file pone.0103714.s005.doc]

**Figure S5**. **Distribution of distinct genotypes for the *RP2* onshore tandem GAAA repeat** (**A**) **and *AR* tandem CAG repeat** (**B**) **loci in the first population subset** (**n = 60 Brazilian females**). The number of females of each genotype, the frequency of each allele and the heterozygosity rate are shown. The allele names (highlighted in yellow) are the lengths in base pairs of each fluorescence peak

(**A**)

| **Total** | **60** |  |  |  | | | |  |  |  |  |  | | |  |
| --- | --- | --- | --- | --- | --- | --- | --- | --- | --- | --- | --- | --- | --- | --- | --- |
| **Homos** | **9** | **15**.**0%** |  |  | | | |  |  |  |  |  | | | |
| **Hets** | **51** | **85**.**0%** |  |  |  |  |  |  |  |  |  |  |  |  |  |
| Frequency | | Allele | 350 | 354 | 357 | 361 | 364 | 368 | 372 | 376 | 379 | 383 | 387 | 391 |  |
| 0.8% | 1 | 350 |  |  |  |  |  |  |  |  |  |  |  |  |  |
| 0.8% | 1 | 354 |  |  |  |  |  |  |  |  |  |  |  |  |  |
| 3.3% | 4 | 357 |  |  |  |  |  |  |  |  |  |  |  |  |  |
| 2.5% | 3 | 361 |  |  |  |  |  |  |  |  |  |  |  |  |  |
| 11.7% | 14 | 364 |  |  |  |  |  |  |  |  |  |  |  |  |  |
| 21.7% | 26 | 368 |  |  | 1 |  | 3 | 5 |  |  |  |  |  |  |  |
| 15.8% | 19 | 372 | 1 |  | 1 |  | 4 | 4 | 1 |  |  |  |  |  |  |
| 25.8% | 31 | 376 |  |  | 1 | 3 | 3 | 6 | 5 | 3 |  |  |  |  |  |
| 10.8% | 13 | 379 |  | 1 | 1 |  | 3 | 2 | 2 | 4 |  |  |  |  |  |
| 4.2% | 5 | 383 |  |  |  |  | 1 |  |  | 2 |  |  |  |  |  |
| 1.7% | 2 | 387 |  |  |  |  |  |  |  | 1 |  | 1 |  |  |  |
| 0.8% | 1 | 391 |  |  |  |  |  |  |  |  |  | 1 |  |  |  |

(**B**)

| **Total** | **60** |  |  |  | | | |  |  |  |  |  | | |  |  |  |  |
| --- | --- | --- | --- | --- | --- | --- | --- | --- | --- | --- | --- | --- | --- | --- | --- | --- | --- | --- |
| **Homos** | **9** | **15**.**0%** |  |  | | | |  |  |  |  |  | | | |  |  |  |
| **Hets** | **51** | **85**.**0%** |  |  |  |  |  |  |  |  |  |  |  |  |  |  |  |  |
| Frequency | | Allele | 209 | 212 | 215 | 218 | 221 | 224 | 227 | 230 | 233 | 236 | 239 | 242 | 245 | 248 | 251 | 260 |
| 0.8% | 1 | 209 |  |  |  |  |  |  |  |  |  |  |  |  |  |  |  |  |
| 2.5% | 3 | 212 |  |  |  |  |  |  |  |  |  |  |  |  |  |  |  |  |
| 4.2% | 5 | 215 |  |  |  |  |  |  |  |  |  |  |  |  |  |  |  |  |
| 8.3% | 10 | 218 |  | 1 | 1 |  |  |  |  |  |  |  |  |  |  |  |  |  |
| 9.2% | 11 | 221 |  |  |  | 1 | 1 |  |  |  |  |  |  |  |  |  |  |  |
| 6.7% | 8 | 224 |  |  | 1 | 1 |  |  |  |  |  |  |  |  |  |  |  |  |
| 23.3% | 28 | 227 |  |  |  | 4 | 2 | 3 | 4 |  |  |  |  |  |  |  |  |  |
| 9.2% | 11 | 230 |  |  | 1 |  | 3 | 1 | 1 | 2 |  |  |  |  |  |  |  |  |
| 10.0% | 12 | 233 | 1 | 1 |  | 1 |  |  | 5 |  |  |  |  |  |  |  |  |  |
| 8.3% | 10 | 236 |  |  |  |  |  | 1 | 2 | 1 | 2 | 1 |  |  |  |  |  |  |
| 5.8% | 7 | 239 |  | 1 | 1 |  | 3 |  |  |  |  | 1 |  |  |  |  |  |  |
| 5.8% | 7 | 242 |  |  |  |  |  | 1 | 1 |  | 1 |  | 1 | 1 |  |  |  |  |
| 3.3% | 4 | 245 |  |  | 1 |  |  |  | 2 |  | 1 |  |  |  |  |  |  |  |
| 0.8% | 1 | 248 |  |  |  |  |  |  |  |  |  | 1 |  |  |  |  |  |  |
| 0.8% | 1 | 251 |  |  |  | 1 |  |  |  |  |  |  |  |  |  |  |  |  |
| 0.8% | 1 | 260 |  |  |  |  |  |  |  |  |  |  |  | 1 |  |  |  |  |
