# Supplementary material for: 5meCpG Epigenetic Marks Neighboring a Primate-Conserved Core Promoter Short Tandem Repeat Indicate X-Chromosome Inactivation
Source: PLoS One. 2014 Jul 31;9(7):e103714. doi: 10.1371/journal.pone.0103714 (PMC4117532; doi:10.1371/journal.pone.0103714)
Supplement: Figure S6 — Distribution of distinct genotypes for the RP2 onshore tandem GAAA repeat (A) and AR tandem CAG repeat (B) loci in the first population subset (n = 60 Dutch females). (DOC) [file pone.0103714.s006.doc]

Figure S6. Distribution of distinct genotypes for the *RP2* onshore tandem GAAA repeat (A) and *AR* tandem CAG repeat (B) loci in the first population subset (n = 60 Dutch females). The number of females of each genotype, the frequency of each allele and the heterozygosity rate are shown. The *AR* primer pair used to genotype the Dutch females is external to the primer pair used to genotype the Brazilian and Argentinean samples (see the Materials and Methods section). For this reason, the *AR* tandem CAG repeat PCR products (alleles) are 44-bp longer in the Dutch females than in the Brazilian and Argentinean females. For example, *in silico* amplification using the NC_000023 reference assembly at the UCSC Genome Browser ([**http://genome.ucsc.edu**](http://genome.ucsc.edu/)) [1] with the external primer pair yields a 288-bp allele, whereas using the internal primers, the reaction yields a 244-bp allele. The allele names (highlighted in yellow) are the lengths in base pairs of each fluorescence peak.

(**A**)

| **Total** | **60** |  |  |  | | | |  |  |  |  |
| --- | --- | --- | --- | --- | --- | --- | --- | --- | --- | --- | --- |
| **Homos** | **16** | **26**.**7%** |  |  | | | |  |  |  |  |
| **Hets** | **44** | **73**.**3%** |  |  |  |  |  |  |  |  |  |
| Frequency | | Allele | 350 | 357 | 361 | 364 | 368 | 372 | 376 | 379 | 383 |
| 0.8% | 1 | 350 |  |  |  |  |  |  |  |  |  |
| 2.5% | 3 | 357 |  |  |  |  |  |  |  |  |  |
| 3.3% | 4 | 361 |  |  |  |  |  |  |  |  |  |
| 11.7% | 14 | 364 |  | 1 | 1 | 2 |  |  |  |  |  |
| 18.3% | 22 | 368 |  | 2 | 1 | 3 | 2 |  |  |  |  |
| 20.8% | 25 | 372 | 1 |  | 2 | 1 | 6 | 4 |  |  |  |
| 23.3% | 28 | 376 |  |  |  | 1 | 5 | 3 | 6 |  |  |
| 11.7% | 14 | 379 |  |  |  | 3 |  | 3 | 2 | 2 |  |
| 7.5% | 9 | 383 |  |  |  |  | 1 | 1 | 5 | 2 |  |

(**B**)

| **Total** | **60** |  |  |  | | | |  |  |  |  |  | | |  |  |
| --- | --- | --- | --- | --- | --- | --- | --- | --- | --- | --- | --- | --- | --- | --- | --- | --- |
| **Homos** | **8** | **13**.**3%** |  |  | | | |  |  |  |  |  | | | |  |
| **Hets** | **52** | **86**.**7%** |  |  |  |  |  |  |  |  |  |  |  |  |  |  |
| Frequency | | Allele | 262 | 265 | 268 | 271 | 274 | 277 | 280 | 283 | 286 | 289 | 292 | 295 | 298 | 301 |
| 2.5% | 3 | 262 |  |  |  |  |  |  |  |  |  |  |  |  |  |  |
| 5.0% | 6 | 265 | 1 |  |  |  |  |  |  |  |  |  |  |  |  |  |
| 5.8% | 7 | 268 | 1 |  |  |  |  |  |  |  |  |  |  |  |  |  |
| 9.2% | 11 | 271 |  |  |  |  |  |  |  |  |  |  |  |  |  |  |
| 13.3% | 16 | 274 |  |  |  | 3 | 1 |  |  |  |  |  |  |  |  |  |
| 25.8% | 31 | 277 |  |  | 1 | 4 | 4 | 6 |  |  |  |  |  |  |  |  |
| 10.8% | 13 | 280 |  | 1 | 1 | 2 | 2 | 3 | 1 |  |  |  |  |  |  |  |
| 5.0% | 6 | 283 |  | 1 |  | 2 |  | 1 |  |  |  |  |  |  |  |  |
| 12.5% | 15 | 286 | 1 | 2 | 4 |  |  | 3 | 2 | 1 |  |  |  |  |  |  |
| 3.3% | 4 | 289 |  | 1 |  |  | 3 |  |  |  |  |  |  |  |  |  |
| 3.3% | 4 | 292 |  |  |  |  | 1 | 1 |  |  | 2 |  |  |  |  |  |
| 1.7% | 2 | 295 |  |  |  |  | 1 | 1 |  |  |  |  |  |  |  |  |
| 0.8% | 1 | 298 |  |  |  |  |  |  |  | 1 |  |  |  |  |  |  |
| 0.8% | 1 | 301 |  |  |  |  |  | 1 |  |  |  |  |  |  |  |  |

**References cited in this supporting information**

1. Kent WJ, Sugnet CW, Furey TS, Roskin KM, Pringle TH, et al. (2002) The human genome browser at UCSC. Genome Res 12: 996-1006.
