# Supplementary material for: 5meCpG Epigenetic Marks Neighboring a Primate-Conserved Core Promoter Short Tandem Repeat Indicate X-Chromosome Inactivation
Source: PLoS One. 2014 Jul 31;9(7):e103714. doi: 10.1371/journal.pone.0103714 (PMC4117532; doi:10.1371/journal.pone.0103714)
Supplement: Figure S7 — Genotypes for the RP2 onshore tandem GAAA repeat (A) and AR tandem CAG repeat (B) loci in the third population subset (n = 46 Brazilian females and n = 4 Argentinean females), consisting of women with known AR tandem CAG repeat 5meC allele-specific profiles and, hence, known XCI ratios. (DOC) [file pone.0103714.s007.doc]

**Figure S7**. **Genotypes for the *RP2* onshore tandem GAAA repeat** (**A**) **and *AR* tandem CAG repeat** (**B**) **loci in the third population subset** (**n = 46 Brazilian females and n = 4 Argentinean females**), **consisting of women with known *AR* tandem CAG repeat 5^me^C allele-specific profiles and**, **hence**, **known XCI ratios**. The allele names (highlighted in yellow) are the lengths in base pairs of each fluorescence peak.

(**A**)

| **Total** | **50** |  |  |  | | | |  |  |  |  |  | | |
| --- | --- | --- | --- | --- | --- | --- | --- | --- | --- | --- | --- | --- | --- | --- |
| **Hets** | **50** | **100%** |  |  |  |  |  |  |  |  |  |  |  |  |
| Frequency | | Allele | 346 | 350 | 354 | 357 | 361 | 364 | 368 | 372 | 376 | 379 | 383 | 387 |
| 1.0% | 1 | 346 |  |  |  |  |  |  |  |  |  |  |  |  |
| 1.0% | 1 | 350 |  |  |  |  |  |  |  |  |  |  |  |  |
| 2.0% | 2 | 354 |  |  |  |  |  |  |  |  |  |  |  |  |
| 4.0% | 4 | 357 |  |  |  |  |  |  |  |  |  |  |  |  |
| 8.0% | 8 | 361 |  |  | 1 |  |  |  |  |  |  |  |  |  |
| 12.0% | 12 | 364 |  |  | 1 | 1 |  |  |  |  |  |  |  |  |
| 20.0% | 20 | 368 | 1 |  |  | 1 | 2 | 2 |  |  |  |  |  |  |
| 25.0% | 25 | 372 |  | 1 |  | 2 | 2 | 3 | 7 |  |  |  |  |  |
| 11.0% | 11 | 376 |  |  |  |  | 1 | 1 | 5 | 4 |  |  |  |  |
| 11.0% | 11 | 379 |  |  |  |  | 1 | 2 | 2 | 5 |  |  |  |  |
| 4.0% | 4 | 383 |  |  |  |  | 1 | 1 |  | 1 |  | 1 |  |  |
| 1.0% | 1 | 387 |  |  |  |  |  | 1 |  |  |  |  |  |  |

(**B**)

| **Total** | **50** |  |  |  | | | |  |  |  |  |  | | |  |  |  |  |  |
| --- | --- | --- | --- | --- | --- | --- | --- | --- | --- | --- | --- | --- | --- | --- | --- | --- | --- | --- | --- |
| **Hets** | **50** | **100%** |  |  |  |  |  |  |  |  |  |  |  |  |  |  |  |  |  |
| Frequency | | Allele | 206 | 209 | 212 | 215 | 218 | 221 | 224 | 227 | 230 | 233 | 236 | 239 | 242 | 245 | 248 | 251 | 260 |
| 1.0% | 1 | 206 |  |  |  |  |  |  |  |  |  |  |  |  |  |  |  |  |  |
| 1.0% | 1 | 209 |  |  |  |  |  |  |  |  |  |  |  |  |  |  |  |  |  |
| 1.0% | 1 | 212 |  |  |  |  |  |  |  |  |  |  |  |  |  |  |  |  |  |
| 4.0% | 4 | 215 |  |  |  |  |  |  |  |  |  |  |  |  |  |  |  |  |  |
| 8.0% | 8 | 218 |  |  |  |  |  |  |  |  |  |  |  |  |  |  |  |  |  |
| 11.0% | 11 | 221 |  |  |  | 1 |  |  |  |  |  |  |  |  |  |  |  |  |  |
| 13.0% | 13 | 224 | 1 | 1 |  |  | 2 | 2 |  |  |  |  |  |  |  |  |  |  |  |
| 10.0% | 10 | 227 |  |  |  | 1 | 1 | 1 | 2 |  |  |  |  |  |  |  |  |  |  |
| 12.0% | 12 | 230 |  |  |  |  |  | 2 | 1 | 3 |  |  |  |  |  |  |  |  |  |
| 7.0% | 7 | 233 |  |  |  | 1 | 2 |  | 1 |  | 1 |  |  |  |  |  |  |  |  |
| 11.0% | 11 | 236 |  |  | 1 | 1 |  | 3 | 2 |  | 1 |  |  |  |  |  |  |  |  |
| 5.0% | 5 | 239 |  |  |  |  |  | 2 |  |  | 3 |  |  |  |  |  |  |  |  |
| 7.0% | 7 | 242 |  |  |  |  | 2 |  | 1 |  |  |  | 3 |  |  |  |  |  |  |
| 4.0% | 4 | 245 |  |  |  |  |  |  |  | 1 | 1 | 1 |  |  |  |  |  |  |  |
| 2.0% | 2 | 248 |  |  |  |  |  |  |  | 1 |  |  |  |  | 1 |  |  |  |  |
| 2.0% | 2 | 251 |  |  |  |  | 1 |  |  |  |  | 1 |  |  |  |  |  |  |  |
| 1.0% | 1 | 260 |  |  |  |  |  |  |  |  |  |  |  |  |  | 1 |  |  |  |
