# Supplementary material for: 5meCpG Epigenetic Marks Neighboring a Primate-Conserved Core Promoter Short Tandem Repeat Indicate X-Chromosome Inactivation
Source: PLoS One. 2014 Jul 31;9(7):e103714. doi: 10.1371/journal.pone.0103714 (PMC4117532; doi:10.1371/journal.pone.0103714)
Supplement: Figure S8 — The RP2 onshore tandem GAAA repeat locus is conserved in primates. (DOC) [file pone.0103714.s008.doc]

**Figure S8**. **The *RP2* onshore tandem GAAA repeat locus is conserved in primates**. A graphic display (available at the UCSC Genome Browser ([http://genome.ucsc.edu](http://genome.ucsc.edu/)) [1]) of multiple alignments and conservation across the *RP2* promoter-bond region in 100 vertebrate genomic reference sequences. The *in silico* 383-bp human genome reference amplimer (hg19 coordinates: chrX:46695746+46696128), encompassing the *RP2* onshore tandem GAAA repeat locus, was used as a query sequence (upper track, represented by solid blocks where the primers (forward primer: 5' TGACATAGCGAGACCCTGTG 3'; reverse primer: 5' GTGGTGGGTTCTCTAGCTG 3') are positioned with a double line indicating the sequence between them). The amplimer encompasses the *RP2* onshore tandem GAAA simple repeat locus, which was annotated using the RepeatMasker program (<http://www.repeatmasker.org/>). The alignment and conservation analyses were carried out online using the Multiz [2] comparative genomics tool available at the UCSC Genome Browser, and viewed in pack (Multiz) and Basewise conservation (phyloP) configurations. Genomic Evolutionary Rate Profiling (GERP) scores for mammalian alignments correspond to position-specific estimates of evolutionary constraint using maximum likelihood evolutionary rate estimation. Public tracks used: Vertebrate Multiz Alignment & Conservation (100 Species), Primate Genomes, Chain and Net Alignments, Non-placental Vertebrate Genomes, Chain and Net Alignments, GERP Scores for Mammalian Alignments.,,

,,


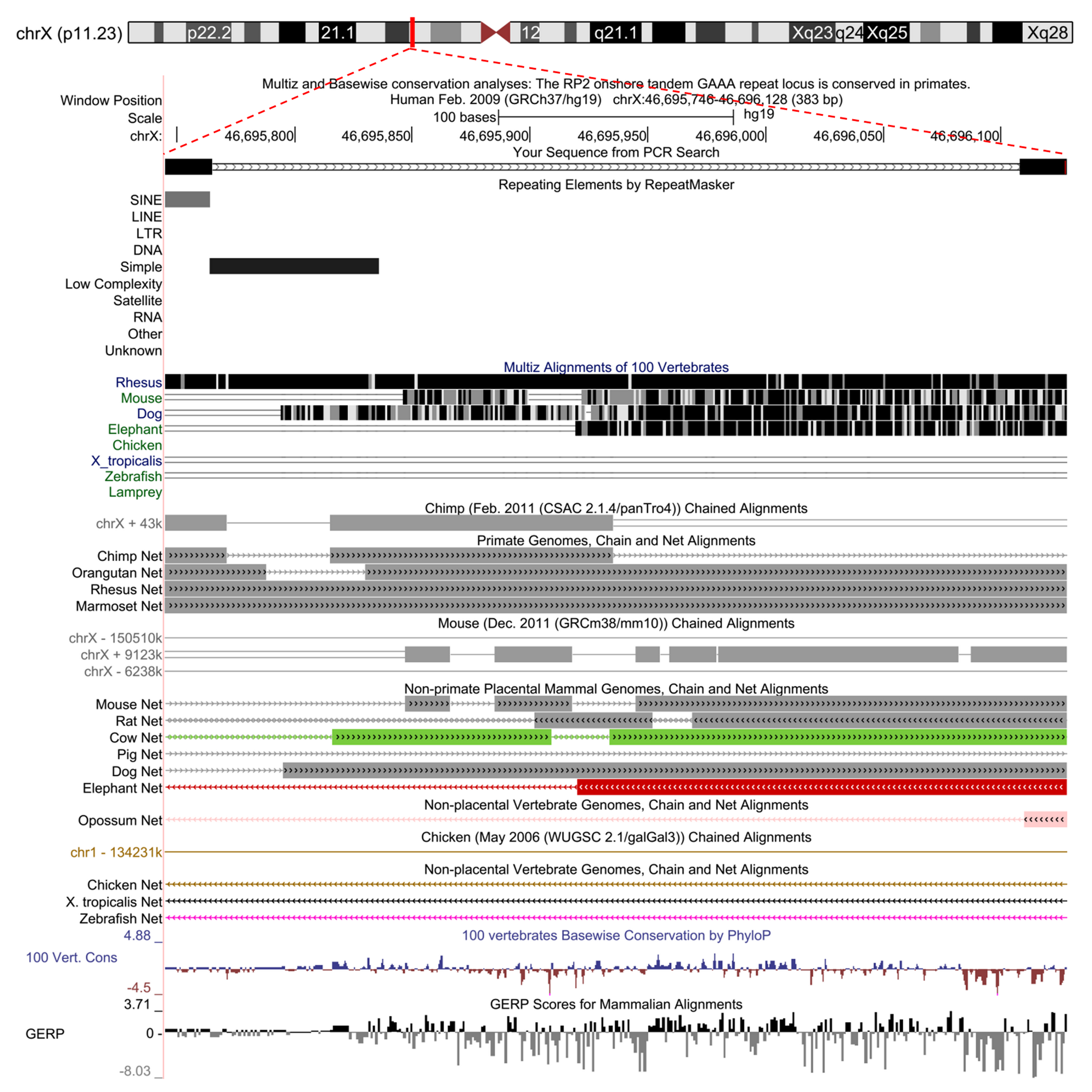


**References**

1. Kent WJ, Sugnet CW, Furey TS, Roskin KM, Pringle TH, et al. (2002) The human genome browser at UCSC. Genome Res 12: 996-1006.

2. Blanchette M, Kent WJ, Riemer C, Elnitski L, Smit AF, et al. (2004) Aligning multiple genomic sequences with the threaded blockset aligner. Genome Res 14: 708-715.
