# Supplementary material for: 5meCpG Epigenetic Marks Neighboring a Primate-Conserved Core Promoter Short Tandem Repeat Indicate X-Chromosome Inactivation
Source: PLoS One. 2014 Jul 31;9(7):e103714. doi: 10.1371/journal.pone.0103714 (PMC4117532; doi:10.1371/journal.pone.0103714)
Supplement: Figure S9 — Multiple sequence alignment of the RP2 onshore tandem GAAA repeat locus reveals high conservation in primates. (DOC) [file pone.0103714.s009.doc]

**Figure S9**. **Multiple sequence alignment of the *RP2* onshore tandem GAAA repeat locus reveals high conservation in primates**. The locations of the forward and reverse primers used for genotyping the *RP2* onshore tandem GAAA repeat in humans are highlighted in red and pink, respectively. The tandem GAAA repeat is highlighted in yellow. The sequence alignment was performed with ClustalW [1].

Homo TGACATAGCGAGACCCTGTGAAAGAAAAAGAAAGAAAGAAAGAAAGAAAGAAAGAAAGAA 60

Macaca TGACATAATGAGACCCTGTGAATG--AATGAAAGAAAGAAAGAAAGAAAGAAAGAAAGAA 58

Pongo TGACATAGCGAGACCCTGTGAAAG--------------------AAAAAGAAAGAAAGAA 40

Callithrix AACAAGAGTGAAACTCTGTCTCAA-----------AAAAAAAAAAAAAGAAAAGAAAGAA 49

:...* *. **.** **** :.:. *.**..**********

Homo AGAAAGAAAGAAAGAAAGAAAGAAAGAAAGAGCACAGAAGAGGATTGGGAGGTTATGGGG 120

Macaca AGAAAGAAAGAAAGAAAGAAAGAAAGAGCGAGCACAGAAGAGGATTGTGAGGTTATGGGG 118

Pongo AGATAAAAAG----------------------CACAGAAGAGGATTGGGAGGTTATGGGG 78

Callithrix AGAAAGAAAGAAAAGATAAAA---------AGCACAGAAGAGGATTGGGAGGTTATGGGG 100

***:*.**** *************** ************

Homo TACAATTCTTGAGGGGGTGACCCTGGCCAGGCGATGAGAGGGCGGTAGGAAGGGTGATGA 180

Macaca TACAATTCTTGAGGGGGTGACCCTGGCCAGGCGATGAGAGGGCGGTAGGAAGGGTGATGA 178

Pongo TACAATTTTTGAGGGAGTGACCCTGGCCAGGCGATGAGAGGGCGGTAGGAAGGGTGATGA 138

Callithrix TACAGTTATTGAGGGGGTAACCCTGGCCAGGCAATGAGGGGGTGGTAGGAAGGGTGATAC 160

****.** *******.**.*************.*****.*** ***************..

Homo GGGGAGAAGAGGATTTAGAAATACAAATTTCAGGGTCTTCTTGTGTCAGCGGGAATTTCT 240

Macaca GGGGAGAAGAGGATTTAGTAATACAAATTTCAGGGTCTTCTTGTGTCAGCGGGAATTTCT 238

Pongo GGGGAGAAGACGATTTAGAAATACAAATTTCAGGGTCTTCTTGTGTCAGCGGGAATTTCT 198

Callithrix GGGGAGAAGAGGATTTAGGAATACAAATTTCAGGGTCTTTTTGTGTCCGCAGGAATTTCT 220

********** ******* ******************** *******.**.*********

Homo G-TCCCTCACAACTTTCATCATAAGATAAATCTAATGTTCAACTAGAGATCTCTCCCGCG 299

Macaca GTTCCCTCACAACTTTCCTCACACGATAAA-CTAATGTTCAACTGGAGACCTCTCCCGCG 297

Pongo G-TCCCTCACAACTTTCATCATAAGATAAATCTAATGTTCAACTAGAGACCTCTCCCGCG 257

Callithrix GTTCCCTCACACCTTTCATCATAAGATAAATCTAATGTTCGACTAGAGACCGCTCCCGCG 280

* *********.*****.*** *.****** *********.***.**** * ********

Homo CCTTGAACTTGCAAATTTATGAATCAGGGGCAAAAAAAACCCGGATACCGAGCCTGGCCT 359

Macaca CCTTGAATTTGCAAATTTATGAATCAGAGGCAAAAAAA-CCCGAATGCCGAGCTCGGCCT 356

Pongo CCTTGAACTTGCAAATTTATCAATCGG-GGCAAAAAAAACCCGGATGCCGAGCCTGGCCT 316

Callithrix ACTTGAACTTGCAAATGTATGAACCAGGGGGCAAAAAAACCCGGCTGCCGAGCCTGGCCT 340

.****** ******** *** ** *.* ** .****** ****..*.****** *****

Homo CCCACCAGCTAGAGAACCCACCA 382

Macaca CCCACCAGCTAGAGAGCCCACCA 379

Pongo CCCACCAGCTAGAGAGCCCACCA 339

Callithrix CCCACCAGCAAGAGAGCCCACCA 363

*********:*****.*******

**References**

1. Thompson JD, Higgins DG, Gibson TJ (1994) CLUSTAL W: improving the sensitivity of progressive multiple sequence alignment through sequence weighting, position-specific gap penalties and weight matrix choice. Nucleic Acids Res 22: 4673-4680.
