# Supplementary material for: 5meCpG Epigenetic Marks Neighboring a Primate-Conserved Core Promoter Short Tandem Repeat Indicate X-Chromosome Inactivation
Source: PLoS One. 2014 Jul 31;9(7):e103714. doi: 10.1371/journal.pone.0103714 (PMC4117532; doi:10.1371/journal.pone.0103714)
Supplement: Table S1 — PCR primer sequences used in this study. (DOC) [file pone.0103714.s010.doc]

**Table S1**. **PCR primer sequences used** **in this study**.

| **Target locus** | **Forward** | **Reverse** | **Modification** | **Application in this study** |
| --- | --- | --- | --- | --- |
| *RP2* GAAA repeat (humans) | tgacatagcgagaccctgtg | **G**tggtgggttctctagctgg **^a^** | Forward PET-labeled | QF-PCR **^b^**  Reverse Transcription-PCR |
| *AR* CAG repeat [1] (humans) | gtgcgcgaagtgatccagaa | ccaggaccaggtagcctgtg | Forward FAM-labeled | QF-PCR (Brazilian and Argentinean samples) |
| *AR* CAG repeat [1] (humans) **^c^** | tccagaatctgttccagagcgtgc | gctgtgaaggttgctgttcctcat | Forward FAM-labeled | QF-PCR (Dutch samples) |
| *RP2* GAAA repeat (Marmosets) | ttgcactccagactgggtaa | gcccctggttcatacatttg | Forward FAM-labeled | QF-PCR |
| *GAPDH* (humans) | agatccctccaaaatcaagtg | ggcagagatgatgacccttt | None | Reverse Transcription-PCR |

**^a^**  The non-templated 5’ guanine residue in this reverse primer was added to promote amplimer adenylation by the Taq DNA polymerase [2].

**^b^** Quantitative fluorescent PCR.

**^c^** This primer pair is external to the primer pair used to genotype the Brazilian and Argentinean samples. For this reason, the *AR* tandem CAG repeat PCR products (alleles) are 44-bp longer in Dutch females than in Brazilian and Argentinean females. For example, *in silico* amplification using theNC_000023 reference assembly sequence of the X-chromosome at UCSC Genome Browser ([http://genome.ucsc.edu](http://genome.ucsc.edu/)) [3] with the external primer pair yields a 288-bp allele, whereas using the internal primers, the reaction yields a 244-bp allele.

**References**

1. Araujo A, Ramos ES (2008) Cryptic mosaicism involving a second chromosome X in patients with Turner syndrome. Braz J Med Biol Res 41: 368-372.

2. Hill CR, Kline MC, Coble MD, Butler JM (2008) Characterization of 26 miniSTR loci for improved analysis of degraded DNA samples. J Forensic Sci 53: 73-80.

3. Kent WJ, Sugnet CW, Furey TS, Roskin KM, Pringle TH, et al. (2002) The human genome browser at UCSC. Genome Res 12: 996-1006.
