# Supplementary material for: 5meCpG Epigenetic Marks Neighboring a Primate-Conserved Core Promoter Short Tandem Repeat Indicate X-Chromosome Inactivation
Source: PLoS One. 2014 Jul 31;9(7):e103714. doi: 10.1371/journal.pone.0103714 (PMC4117532; doi:10.1371/journal.pone.0103714)
Supplement: Table S2 — Structure of the RP2 onshore tandem GAAA repeat region in primates. (DOC) [file pone.0103714.s011.doc]

**Table S2**. **Structure of the *RP2* onshore tandem GAAA repeat region in primates**.

| **Species** ^a^ | **Structure of the tandem repeat region** | **NCBI Accession** |
| --- | --- | --- |
| Human | (AAAG)_16_ ^b^ | [NC_000023.10](http://www.ncbi.nlm.nih.gov/nucleotide/224589822?report=genbank&log$=nucltop&blast_rank=2&RID=1U98HRS201R) |
| Rhesus | (AAAG)_14_ | [NC_007878.1](http://www.ncbi.nlm.nih.gov/nucleotide/109158195?report=genbank&log$=nucltop&blast_rank=6&RID=1U98HRS201R) |
| Baboon | (AAAG)_12_GG(AAAG)_3_ | [NC_018172.1](http://www.ncbi.nlm.nih.gov/nucleotide/395728641?report=genbank&log$=nucltop&blast_rank=9&RID=1U98HRS201R) |
| Gibbon | (AAAG)_6_AAAAG(AAAG)_2_ | [NC_019841.1](http://www.ncbi.nlm.nih.gov/nucleotide/429476316?report=genbank&log$=nucltop&blast_rank=4&RID=1U98HRS201R) |
| Bonobo | (AAAG)_5_ | [NW_003870269.1](http://www.ncbi.nlm.nih.gov/nucleotide/393728392?report=genbank&log$=nucltop&blast_rank=7&RID=1U98HRS201R) |
| Chimpanzee | (AAAG)_5_ | [NC_006491.3](http://www.ncbi.nlm.nih.gov/nucleotide/291061354?report=genbank&log$=nucltop&blast_rank=12&RID=1U98HRS201R) |
| Marmoset | (AAAG)AAAAG(AAAG)_5_ | [NC_013918.1](http://www.ncbi.nlm.nih.gov/nucleotide/290467386?report=genbank&log$=nucltop&blast_rank=11&RID=1U98HRS201R) |
| Gorilla | (AAAG)_3_ATAG(AAAG)_2_ | [NC_018447.1](http://www.ncbi.nlm.nih.gov/nucleotide/401623065?report=genbank&log$=nucltop&blast_rank=5&RID=1U98HRS201R) |
| Orangutan | (AAAG)_4_ | [NC_012614.1](http://www.ncbi.nlm.nih.gov/nucleotide/241864894?report=genbank&log$=nucltop&blast_rank=8&RID=1U98HRS201R) |
| Squirrel monkey | (AAAG)_2_ACG(AAAG)AA(AAAG)_3_[...//...](AG)_2_AA(AG)_8_AC(AG)_8_AC(AG)_6_ | [NW_003943719.1](http://www.ncbi.nlm.nih.gov/nucleotide/395722533?report=genbank&log$=nucltop&blast_rank=10&RID=1U98HRS201R) |

^a^ In the downward order: *Homo sapiens*, *Macaca mulatta*, *Papio anubis*, *Nomascus leucogenys*, *Pan paniscus*, *Pan troglodytes, Callithrix jacchus*, *Gorilla gorilla gorilla*, *Pongo abelii*, *Saimiri boliviensis boliviensis*.

### ^b^ The numbers indicate the lengths of the arrays in tandem uninterrupted repeat units.
